# Genetic Mapping of Millions of SNPs in Safflower (*Carthamus tinctorius* L.) via Whole-Genome Resequencing

**DOI:** 10.1534/g3.115.026690

**Published:** 2016-05-24

**Authors:** John E. Bowers, Stephanie A. Pearl, John M. Burke

**Affiliations:** * Department of Plant Biology, Miller Plant Sciences, University of Georgia, Athens, Georgia 30602

**Keywords:** WGS map, genetic map, safflower

## Abstract

Accurate assembly of complete genomes is facilitated by very high density genetic maps. We performed low-coverage, whole-genome shotgun sequencing on 96 F_6_ recombinant inbred lines (RILs) of a cross between safflower (*Carthamus tinctorius* L.) and its wild progenitor (*C. palaestinus* Eig). We also produced a draft genome assembly of *C. tinctorius* covering 866 million bp (∼two-thirds) of the expected 1.35 Gbp genome after sequencing a single, short insert library to ∼21 × depth. Sequence reads from the RILs were mapped to this genome assembly to facilitate SNP identification, and the resulting polymorphisms were used to construct a genetic map. The resulting map included 2,008,196 genetically located SNPs in 1178 unique positions. A total of 57,270 scaffolds, each containing five or more mapped SNPs, were anchored to the map. This resulted in the assignment of sequence covering 14% of the expected genome length to a genetic position. Comparison of this safflower map to genetic maps of sunflower and lettuce revealed numerous chromosomal rearrangements, and the resulting patterns were consistent with a whole-genome duplication event in the lineage leading to sunflower. This sequence-based genetic map provides a powerful tool for the assembly of a low-cost draft genome of safflower, and the same general approach is expected to work for other species.

Genetic linkage maps have long served as powerful tools for the study of genome structure and the genetic basis of trait variation in both plants and animals. The utility of such maps is largely determined by the number of loci and the accuracy and precision with which they have been placed. Initially, genetic maps were based on small numbers of segregating, single gene morphological traits, and the resulting linkage groups had very few loci (*e.g.*, Sturtevant 1913). Over time, it became possible to produce genotypic data from indirectly observed genetic variants, such as isozymes (McKusick and Ruddle 1977). Genetic linkage maps remained sparse, however, until the advent of DNA-based techniques in the 1980s (Botstein *et al.* 1980), which facilitated the more efficient generation of genotypic data. By testing many DNA samples at a time via RFLP or PCR assays, it became possible to produce genetic maps of hundreds or even a few thousand loci. More recently, this paradigm of testing many samples at once for one or a few loci per assay reversed to the simultaneous interrogation of individual samples for hundreds or thousands of loci on a DNA chip (Beattie *et al.* 1995; Bowers *et al.* 2012a,b; Truco *et al.* 2013), effectively reducing the lab work needed to generate genetic maps containing thousands of loci to a few days.

The rapidly falling cost of DNA sequencing has enabled new, low cost options for the generation of genetic mapping data. By sequencing a targeted subset of the whole-genome, such as sequences that flank restriction enzyme cut sites, researchers have been able to generate sequence-based genetic maps with tens of thousands of loci from sequence data produced in a few days. This general technique, known as genotyping-by-sequencing (GBS; Elshire *et al.* 2011) has revolutionized genetic mapping research. But even with these much greater marker densities, more loci are often desired to thoroughly characterize complex genomes. Here, we describe an approach for the construction of ultradense, sequence-based genetic maps using low coverage, whole-genome shotgun (WGS) sequencing data from a set of recombinant inbred lines (RILs). As we near the age of the $1000 genome and the $100,000 analysis (Mardis 2010), it has become increasingly obvious that the development of new approaches for large-scale assembly and analysis, not raw sequencing costs, will drive future breakthroughs in cost and efficiency. Our work represents a step in that direction.

The generation of a genetic map from the WGS sequencing of segregating progeny presents unique computational challenges. While WGS sequencing has the potential to reveal millions of mappable polymorphisms (three or four orders of magnitude higher than previously practical), the resulting datasets present unique computational challenges as the number of possible locus orders corresponds to the factorial of the number of loci. Low coverage sequencing poses additional complications, as many individuals will be missing data at a substantial fraction of all loci. Moreover, heterozygous genotypes will often be genotyped incorrectly, though this latter problem can be minimized via the use of an inbred mapping population. Finally, due to the very large number of loci tested, errors in sequencing and read placement will result in a large number of erroneous data points, even if the overall rate of such errors is low. Thus, the primary challenge when using WGS data for genetic mapping is determining how to best use the data, as opposed to generating the data in the first place.

While the general approach of using WGS sequencing data for genetic mapping has been previously implemented (*e.g.*, Frazer *et al.* 2007; Huang *et al.* 2009; Renaut *et al.* 2013; Wijnker *et al.* 2013; Mascher *et al.* 2013; Ariyadasa *et al.* 2014; Hahn *et al.* 2014; Chapman *et al.* 2015), it has only recently been applied to genomes without existing chromosome-level assemblies. The limited use of WGS sequencing for mapping previously unexplored genomes likely relates to the aforementioned challenges, and the fact that an available genome assembly avoids many of the computational challenges associated with marker ordering. In this paper, we describe the construction of a sequence-based genetic map of the safflower (*Carthamus tinctorius* L.) genome and its use to explore patterns of genome evolution across the Compositae, which is the largest family of flowering plants and includes other economically important species such as lettuce and sunflower.

Safflower has a long history of human use, with archeological remains of *Carthamus* spp. dating back to 7500 BC having been found at sites in Syria (Marinova and Riehl 2009). From there, safflower cultivation is thought to have spread to Egypt, the Aegean, and into southeastern Europe. In ancient times, safflower floral extracts were commonly used as both red and yellow dyes for textiles; hence, the species name *tinctorius*, derived from the Latin *tinctus* (dyed, stained, or tinged), which means ‘used in dyeing.’ Safflower seeds have also been directly consumed on a limited basis, dried and ground flowers have been used in cooking in place of saffron, and it is sometimes grown for medicinal uses. Commercialization of safflower in the Americas commenced in the 1950s, where it has primarily been grown as an oilseed crop, as a source of birdseed, and as an ornamental (Smith 1985; Emongor 2010). Worldwide, safflower is planted on ∼1 million hectares/yr with a total production value of ∼$260 million (http://faostat.fao.org/). It is also notable that safflower is well-adapted to growth in moisture-limited environments and can tolerate saline soils. As such, it can be grown on marginal agricultural lands that are suitable for few other crops (Ghamarnia and Gholamian 2013). Despite this relatively low overall value, it has been suggested that the improvement of minor crops such as safflower could help to provide food security in the face of climate change and an ever-increasing global population (Amini *et al.* 2014).

Because safflower was originally domesticated for an entirely different purpose (*i.e.*, as a source of dye) and has been subjected to relatively limited modern breeding efforts, it seems likely that selection has unlocked only a small portion of its potential as an oilseed crop. To accelerate the ongoing improvement of safflower, we sought to develop an ultradense, sequence-based genetic map that could be used to guide the assembly of the safflower genome. This resource has the potential to facilitate molecular breeding efforts and, when combined with available mapping resources from other species within the Compositae (Kane *et al.* 2011; Bowers *et al.* 2012a; Truco *et al.* 2013), can also be used to investigate patterns of genome evolution within one of the world’s most important flowering plant families.

## Materials and Methods

### Plant materials and draft genome assembly

The initial production of the mapping population was described by Pearl *et al.* (2014). Briefly, a single individual of safflower (cv AC Sunset; PI 592391) was crossed with a single individual of its wild progenitor, *C. palaestinus* (PI 235663). The F_1_ seeds from this cross were then planted, grown to maturity, and self-pollinated to produce the F_2_ generation. The resulting F_2_ lineages were then repeatedly grown and self-pollinated to produce F_6_ RILs. DNA was extracted from each of 96 RILs and five plants from the same seed packets as each of the mapping parents. The individual samples were then barcoded, combined into a single mix, and sequenced on all eight lanes of a single flow cell of an Illumina Hi-Seq (GenBank PRJNA 313950). A genome assembly of the mostly homozygous *C. tinctorius* AC Sunset inbred line was made but, due to the difficulties of genome assembly for heterozygous individuals, the same was not done for the outbred *C. palaestinus*.

We produced a total of 4.7 × 10^9^ bp of sequence data using 100 bp paired-end sequencing reads with an average insert size of 306 bp. Each RIL was thus sequenced to an average of 2.26 × (range 0.92 × – 4.44 ×) coverage of the estimated 1350 Mbp genome size of the safflower genome (Garnatje *et al.* 2006), while the parents were sequenced to a greater depth. Sequence reads from the homozygous AC Sunset parent totaled ∼21.3 × coverage of the estimated genome size, and a draft genome was assembled from these reads using the program SOAPdenovo2 (Luo *et al.* 2012) at K = 63, followed by gap filling using SOAP GapCloser.

### Linkage map construction

Sequence reads from the RILs were mapped onto the draft AC Sunset assembly with Bowtie 2 (Langmead and Salzberg 2012) and processed using SAMtools (Li *et al.* 2009). Genotype scores for individuals at each SNP were computed from LOD values by assuming homozygous genotypes whenever possible (LOD > 0), not the automatic genotype calls produced by SAMtools. The reason for this is that the assumptions of Hardy-Weinberg equilibrium that are used to assign genotypes in SAMtools do not apply to the RILs studied; with RILs, heterozygous genotypes are far less likely than would otherwise be expected. For an F_6_ RIL mapping population, the expected segregation ratio would be 31:2:31 for the AA, AB, and BB genotypes, respectively. Loci that were heterozygous in more than 15% of the RILs were discarded as they likely corresponded to multi-copy sequences. SNPs that segregated with minor allele frequency (MAF) of less than 5% were also discarded, as they were most likely artifacts due to sequencing errors or incorrect placement of sequence reads against the assembly. The final thresholds on MAF and heterozygosity were set empirically based on iterative creation of draft maps with less stringent thresholds. This is because, while the expected levels can be estimated, actual results for a map depend on segregation distortion in the mapping population, which can be highly unpredictable and variable. The observed MAF minimum for haplotypes included in the drafts map was 12.5%, with an observed maximum heterozygosity of 10.5%. The thresholds used for individual SNPs were more liberal than the observed limits on the draft maps to account for sequencing errors and missing data affecting individual SNPs. SNPs with > 50% missing data were also discarded.

As several million SNPs still remained after the above steps, only SNPs with quality scores from SAMtools function Mpileup equal to the 999 maximum score were used. In the next step, SNPs from the same sequence scaffold were merged together to create a consensus scaffold-level haplotype. By constructing a consensus haplotype from all SNPs on a given scaffold, we were able to use low coverage data to unambiguously determine genotypes for a given genetic region, including heterozygous genotypes, and sequencing errors could then be corrected *vs.* the consensus. The resulting scaffold haplotypes were then used to assemble a consensus template map using Microsoft Excel as described by Bowers *et al.* (2012a), beginning with scaffolds containing >50 SNPs to simplify the ordering problem. The somewhat unorthodox approach of using spreadsheet software for genetic map ordering is outlined in Supplemental Material, File S1. The template map was assembled by sorting data columns, where columns correspond to individuals and rows correspond to the consensus SNP haplotypes. This approach provides a simplified and rapid means for ordering a genetic map when most of the genotypic patterns are present in the data (Bowers *et al.* 2012a). The reason that this approach works so well is that, with extremely high marker density, most haplotype patterns are separated just one or a very small number of recombination events from adjacent patterns in the data. Using the assumption that the best genetic map is the one that contains the fewest recombination events, ordering becomes a relatively simple task of placing the most similar multi-locus haplotypes adjacent to each other.

The initial template map was then used to filter all individual SNPs. Individual SNPs that are the product of multi-copy sequences or sequences where the two alleles correspond to different genomic locations would not fit onto the genetic map. All SNPs that showed more than six differences from the most similar location on the template map were discarded as anomalous SNPs. The genotypes of scaffolds were then recomputed with the filtered SNP data, reducing the error rate of the consensus genotype for the scaffold. For comparison to the template, heterozygous loci were assumed to match genotype scores of AA, AB, or BB without flagging them as being different from the template due to the likelihood that heterozygous genotypes at an individual SNP may not be observed due to sequencing depth. The filtered SNPs were then merged by scaffolds and used to manually edit and improve the template map. Removal of the anomalous SNPs from the scaffold prior to determining the consensus improved the accuracy of the scaffold genotypes. This process was repeated, revising the template map each cycle and refiltering the individual SNPs to combine them by scaffolds, until no further changes to the template map were made (15 iterations). The template map was revised and edited with scaffolds containing progressively fewer SNPs, with the final five iterations involving the examination of all scaffolds with ≥ 5 SNPs.

### Analysis of the linkage map

Individual scaffolds that contained multiple SNPs mapping to two different linkage groups or > 10 recombination events apart were identified as mis-assembled scaffolds. Scaffolds containing three or more consecutive SNPs of one homozygous genotype followed by three or more consecutive SNPs of the other homozygous genotype, and in which the template map predicted a crossover event in the corresponding RIL, were inferred to contain recombination breakpoints. DNA sequence from the genome assembly between the SNPs indicating recombination events was extracted, along with 200 bp of flanking sequence on each side. While recombination events also exist on the map between homozygous and heterozygous regions, the difficulty of assigning heterozygous genotypes to individual SNPs due to low sequence coverage prevented precise placement of such events. Therefore, recombination events between heterozygous and homozygous regions were only placed at intervals between scaffolds, not between individual SNPs.

Synteny comparisons to previously published genetic maps of lettuce and sunflower (Bowers *et al.* 2012a; Truco *et al.* 2013) were performed using BLAST to place gene sequences from the other genetic maps onto the safflower draft assembly. The best match for each sequence with e-values < 10^−6^ was then included in a dot plot if the best hit corresponded to a genetically mapped safflower scaffold.

### Data availability

The authors state that all data necessary for confirming the conclusions presented in the article are represented fully within the article.

## Results and Discussion

### Draft genome assembly and SNP identification

Our draft assembly of the safflower genome consisted of 3,254,412 contigs 100 bp or larger that assembled into 2,195,958 scaffolds 100 bp or larger File S2, (http://datadryad.org/resource/doi:10.5061/dryad.635ds; GenBank LUCG00000000 for sequences ≥ 200 bp). The assembly had an overall GC content of 37.3%. The contig N50 size was 368 bp and the scaffold N50 size was 1976 bp (File S3). The total assembled size was 866 Mbp, corresponding to ∼65% of the expected genome size of 1350 Mbp. The incomplete coverage and fragmented nature of our draft assembly were likely due to the use of a single, small insert library at 21 × coverage, combined with the possibility that repetitive genomic sequences failed to assemble or collapse into single contigs. In fact, recommendations for *de novo* WGS assemblies from short reads call for substantially higher levels of sequencing coverage as well as libraries with multiple insert sizes (Gnerre *et al.* 2011). Nonetheless, the suitability of our assembly for polymorphism discovery and genetic mapping demonstrates that even a rudimentary genome assembly can be a valuable resource.

The initial results from calling SNPs after alignment with Bowtie 2 and processing with SAMtools provided 7,822,301 candidate SNPs with a minimum allele frequency > 5%. However, after the removal of SNPs with high levels of heterozygosity, missing data, and/or low quality scores, the number of SNPs was reduced to slightly over two million. With millions of SNPs and missing data rates > 15% at individual SNPs, plus high genotyping error rates at heterozygous sites due to limited sequencing depth, construction of a genetic map by conventional approaches that treat each SNP separately would be exceptionally difficult (Cheema and Dicks 2009). Given the extremely large number of loci, an exhaustive examination of all possible marker orders is all but impossible. Even after merging all SNPs with identical patterns, hundreds of thousands of genotype patterns (due to the segregation of actual polymorphisms as well as sequencing errors and missing data) would still have to be ordered. As such, an alternative approach (outlined above) was required.

### Genetic map description

**Figure 1:**
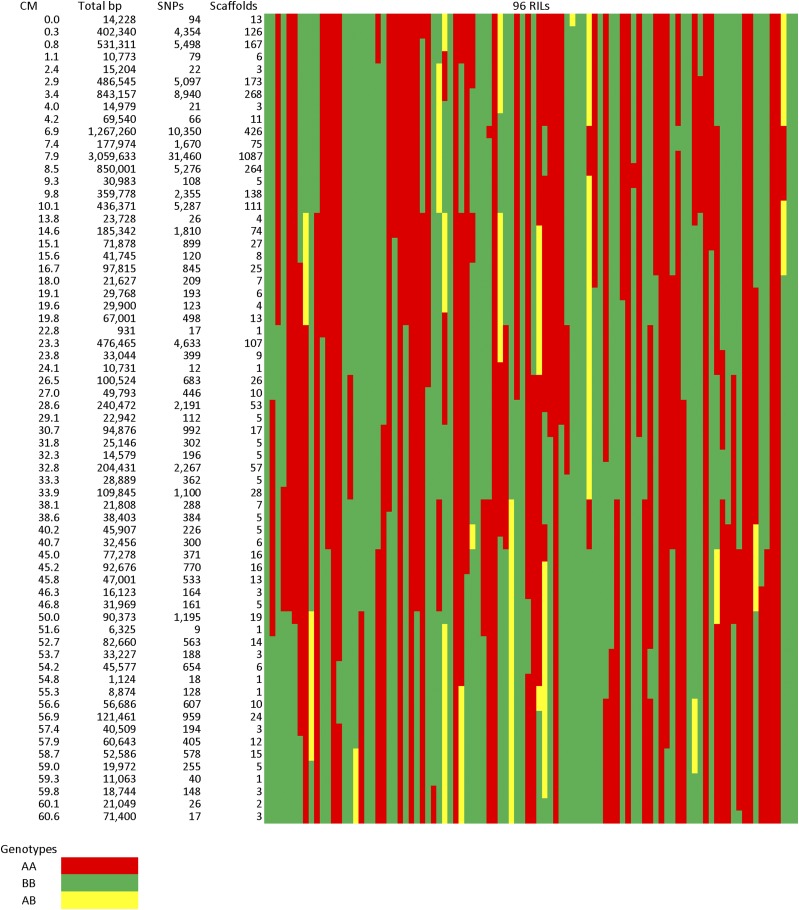
Template genetic map for safflower linkage group 2. A total of 65 unique recombinational patterns were observed across the 96 RILs for this linkage group. The rows correspond to distinct chromosomal locations, and the columns on the right correspond to the 96 RIL lines and the genotype of the RIL at each location. The “A” genotype score corresponds to “AA,” “H” to “AB,” and “B” to “BB,” and the genotypes are color-coded for clarity. A total of 3572 sequence scaffolds containing 11,697,443 bp and 108,293 mapped SNPs were placed on this linkage group. RIL, recombinant inbred line; SNP, single nucleotide polymorphism.

The final template map is located in File S4 (http://datadryad.org/resource/doi:10.5061/dryad.635ds), with an example for one chromosome provided in Figure 1; an overall summary is provided in Table 1. The genetic map of safflower assembled into 12 linkage groups, matching the expected chromosome number. A list of the genetically placed scaffolds and their locations is presented in File S5 (http://datadryad.org/resource/doi:10.5061/dryad.635ds). The template map included 2082 recombination events, of which 544 were transitions from homozygous to heterozygous genotypes, and 1538 were transitions between the two different homozygous genotypes. Given that they occurred across 12 chromosomes, the 2082 recombination events would be expected to produce a total of 2094 genotypic patterns; however, only 1178 unique patterns were actually observed on one or more full scaffold, as several intervals in the map had multiple recombination events between loci. A total of 2,008,196 mapped SNPs File S6 (http://datadryad.org/resource/doi:10.5061/dryad.635ds) could be placed on 57,270 mapped scaffolds that contained 192,589,591 non-N nucleotides. This is an average of one genetically mapped SNP per 95.9 bp, though the actual differences between the two safflower individuals is likely higher than this, as SNPs near repeats or near the ends of scaffolds are less likely to be mapped. The high levels of sequence divergence between the two mapping parents may have prevented accurate placement of sequence reads in more divergent regions.

**Table 1:** Summary characteristics of genetic map by linkage group

Linkage Group	SNPs	Segregation Patterns Observed	Scaffolds Mapped	Length (CM)
1	96,218	80	2750	67.2
2	108,293	65	3572	60.6
3	126,472	109	2983	81.8
4	291,390	59	8808	42.6
5	339,005	117	10,026	83.9
6	308,909	125	9281	99.0
7	108,411	99	2921	77.3
8	112,476	113	2868	102.7
9	75,934	94	1666	96.0
10	139,849	122	3264	89.4
11	77,993	111	2006	86.8
12	223,246	83	7243	72.2
Total	2,008,196	1177	57,388	959.4

SNP, single nucleotide polymorphism.

Despite the high number of SNPs and mapped scaffolds, several gaps remained in the final map. Eight gaps spanning ≥ 5 cM remained in the final map. The largest gap spanned 7.1 cM, corresponding to 14 of the 96 RILs exhibiting recombination events between the flanking loci. These gaps could be regions that are relatively short in physical size, but have high recombination rates (*i.e.*, recombination hotspots; Drouaud *et al.* 2006). Alternatively, they could correspond to regions that largely contain repetitive DNA, which could limit local assembly quality and reduce the frequency of mappable polymorphisms. Finally, it is possible that they represent large genomic regions that are highly similar or identical due to a recent shared ancestor between the two genotypes used as parents in the cross, precluding any SNPs from being mapped in those regions (Bowers *et al.* 2012a).

### Genotyping and assembly errors

For SNPs in mapped scaffolds, the error rates and SNP types could be calculated. The missing data rate for SNPs at individual RILs was 16.9%. For SNPs where the scaffold consensus genotype was heterozygous, 61.2% of the individual SNPs were erroneously scored as being homozygous (*i.e.*, both alleles were not observed). For SNPs where the scaffold consensus genotype was homozygous, 0.5% of the individual SNPs were incorrectly scored as heterozygous, and 0.4% were scored as the other homozygous genotype. For many individual SNPs, exact locations on the genetic map would have been uncertain due to missing data. However, when the SNPs were grouped into scaffolds, 95.2% of the scaffold consensus genotypes fit into a unique position on the map template with no uncertainty. Even when they were not assigned a unique position, most scaffolds could be placed in a narrow window. Indeed, for 99.9% of the SNPs grouped by scaffolds, the genetic map position assigned was a window of 2 cM or less. Of all 2,008,196 genetically mapped SNPs, 64.7% were C/T polymorphisms, 7.5% were C/G polymorphisms, 16.8% were C/A polymorphisms, and 11.0% were A/T polymorphisms. The high frequency of C/T polymorphisms has been noted in many mutation studies, and is likely related to the high frequency of methylated cytosine mutating to thymidine (Youssoufian *et al.* 1986).

Genetic maps such as the one described herein also provide insight into the quality of a genome assembly, particularly with respect to identifying mis-assembled scaffolds while editing and correcting the assembly into chromosome-sized pieces (Paterson *et al.* 2009). In the present study, scaffolds that contained five or more SNPs mapping to two different chromosomes were identified as likely errors in the initial shotgun genome assembly. We found 118 such chimeric scaffolds, corresponding to ∼0.2% of all mapped scaffolds. Of course, the highly fragmented nature of our assembly, with many short scaffolds, reduces the likelihood of chimeric assemblies in this case. In addition to error detection, an ultrahigh density genetic map can be used to place/order scaffolds, as well as to orient the scaffolds that are found to contain recombination events. Although that process would be of limited value with the current assembly, it could greatly improve the quality of a more complete genome assembly.

### Read depth as a filtering tool

**Figure 2:**
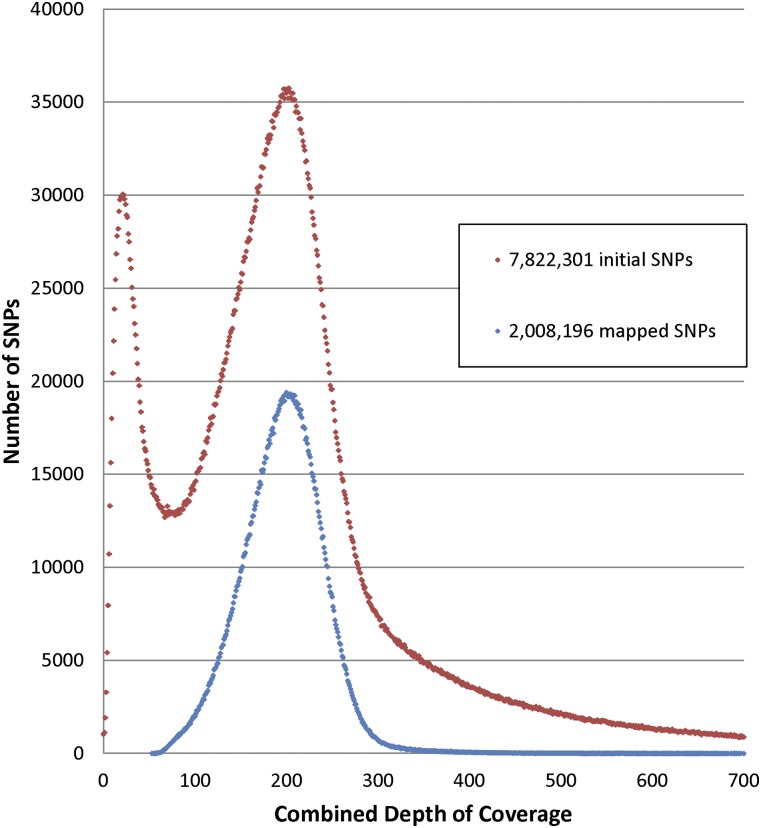
Total mapped sequencing read depth for genetically mapped SNPs, combined across 96 RILs. The modal combined sequencing depth was 200, and 99% of the mapped SNPs were sequenced between 79–358 times, combined across all RILs. RIL, recombinant inbred line; SNP, single nucleotide polymorphism.

The combined sequencing depth across all RILs can be used to identify multi-copy sequences. The combined sequencing depths for the final set of mapped SNPs, as well as the initially identified pool of candidate SNPs, are summarized in Figure 2. For genetically mapped SNPs, the total sequencing depth combined from all RILs fell into a narrow range, while the preliminary candidate SNPs showed a much broader distribution. The combined sequencing depth for 96 RILs of mapped reads had a mode of ∼200 ×. The mapped SNPs fell into a relatively narrow range with 99% of the mapped SNPs having a combined mapped read depth between 82–339 reads. Most genetic polymorphisms that correspond to repetitive sequences were removed in the mapping process, as they would not map to a single location on the template map, or they would show unexpected segregation ratios. For many of the candidate SNPs that we initially identified, the depth of coverage was much broader, with a lower peak of low coverage. Most of these lower coverage SNPs were discarded due to missing data at over 50% of the RILs or low sequence alignment scores. The candidate SNPs also contained a substantial number of SNPs that showed depth of coverage > 400×, most likely corresponding to repetitive sequences. Although we did not do this in the current study, filtering candidate SNPs by combined read depth would be an effective approach to remove sequences corresponding to repetitive DNA.

### Recombination rate variation

Comparisons of whole-genome assemblies to genetic maps in other plant species have revealed that recombination rates vary across the genome (Paterson *et al.* 2009). It is thus not surprising that the number of anchored base pairs per distinct segregation pattern varied greatly in our study. While 44% of the patterns theoretically present in the genetic map (*i.e.*, between observed genotypes) were not observed, some intervals with no recombination were represented by as many as 213,339 SNPs, corresponding to > 10% of all mapped SNPs and totaling 20,497,082 bp of mapped scaffolds. The exact relationship between the local recombination rate and base pair distances can, however, only be approximated by the portion of the genome that is represented by genetically mapped scaffolds, and it is logical to assume that the gene rich, single copy fraction of the genome would both assemble and be genetically mapped more often than the repetitive fraction. It seems likely that the three loci with the largest number of SNPs (located on chromosomes 4, 5, and 6 and harboring 213,339, 135,029, and 85,996 SNPs, respectively – 21.6% of all mapped SNPs combined) are the result of inhibited recombination along a portion of those chromosomes, possibly due to structural rearrangements such as large inversions. In contrast, linkage groups 7, 8, and 9 did not contain any genetic loci with more than 12,815 SNPs. The total number of SNPs that could be mapped per linkage group ranged from 75,934–339,005. The distribution of SNP loci across the map, and the number of scaffolds and base pairs mapped by position, is shown in Figure S1.

**Figure 3:**
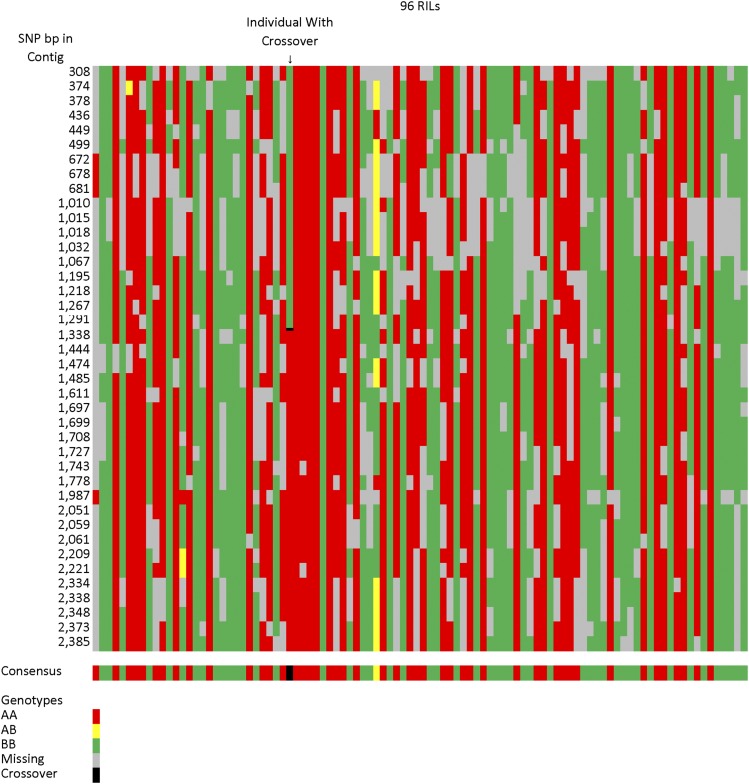
Sample scaffold showing raw genotype scores for 41 SNPs and the consensus genotype for the scaffold. This scaffold shows a recombination event, indicated by a black bar in the consensus, that occurred between 1291–1338 bp. RIL, recombinant inbred line; SNP, single nucleotide polymorphism.

The draft genome assembly allowed us to identify the genomic location of a subset of the observed recombination events. A total of 194 recombination events could be placed within sequence scaffolds, and the location of the recombination events could be localized to regions spanning 7–3748 bp in length (average = 343 bp; see Figure 3 for an example). Additionally, there were four cases in which two recombination events were observed within a single scaffold. These events were 255–409, 407–797, 737–958, and 2400–2686 bp apart, suggesting the existence of recombination hotspots within the safflower genome. The scaffolds with recombination events added an additional 23 genotype patterns that were not present in the genetic map template based on whole scaffolds. As noted above, the exact placement of recombination events between homozygous and heterozygous genotypes was not attempted due to the relatively higher error rate in genotyping heterozygous loci at individual SNPs. Comparison of all the sequences where recombination occurred, plus the flanking 200 bp to each other, revealed no recurring sequence motif that could be detected by BLAST. However, The GC content was slightly lower near recombination events than in the assembly as a whole (36.4% *vs.* 37.3%). While the absolute difference in GC content was slight, it was highly significant (*P* < 0.000000001).

### Comparison to other species

**Figure 4:**
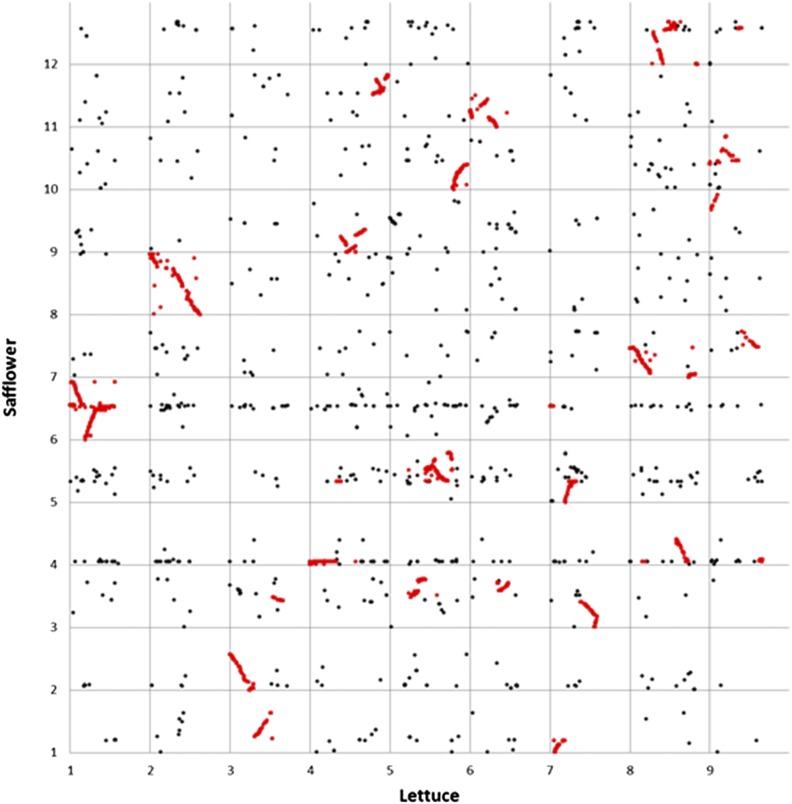
Synteny plot between the lettuce genetic map and the safflower draft genome assembly and genetic map depicting the relative positions of 4614 gene sequences. The genes from 26 syntenic segments highlighted in red contain 78% of all gene sequences on both maps.

Comparisons between the safflower genetic map and draft assembly were made to two previously published maps of lettuce and sunflower (Bowers *et al.* 2012a; Truco *et al.* 2013), which are members of different subfamilies within the Compositae. From the lettuce genetic map, the 12,786 mapped genes included 10,593 (83%) that could be placed on the safflower assembly. Of these, 4614 (44%) matched scaffolds that had been placed on the safflower genetic map. Inspection of Figure 4 reveals that the safflower and lettuce genomes have undergone substantial rearrangement since their most recent common ancestor, with only one pair of linkage groups (LGs; safflower LG 8 and lettuce LG 2) retaining synteny across their entire length. All other safflower and lettuce LGs are partially syntenic to multiple LGs from the other species, except safflower LG 2, which is one of three linkage groups from safflower that contributed to lettuce LG 3. In all, 26 large segments of synteny were observed between lettuce and safflower; these segments contained 3611 of the 4614 genes mapped in both species. This suggests that ∼78% of all genes are in corresponding syntenic positions between the two species. This comparison also suggests that the mapped scaffolds represent a higher than expected fraction of single copy gene sequences in safflower. Indeed, while only 14% of the raw expected genome size of safflower is in genetically mapped scaffolds, 36% of the gene sequences from a related species could be placed on the safflower map.

**Figure 5:**
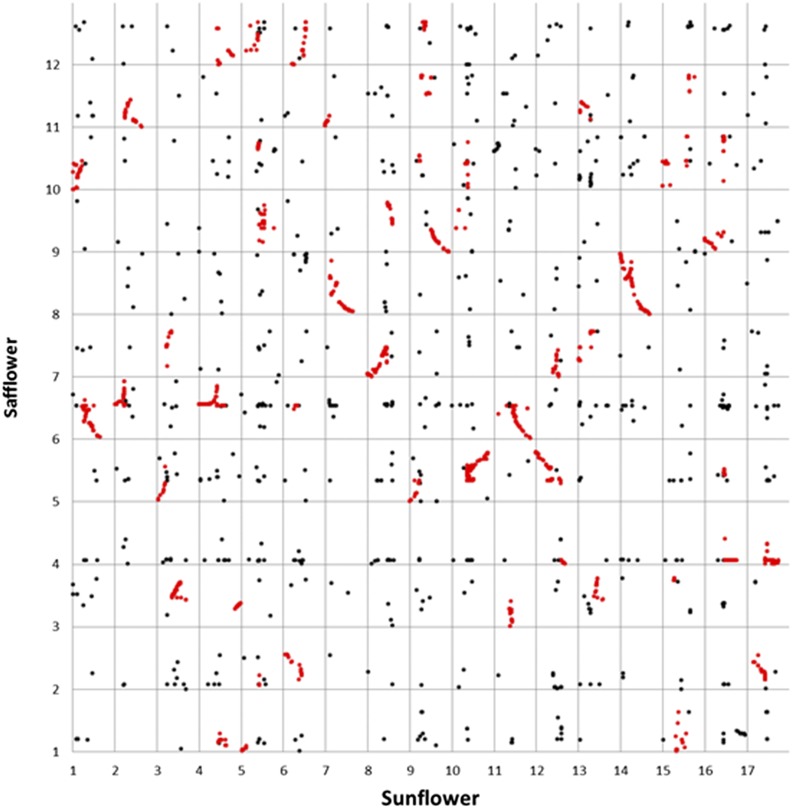
Synteny between the sunflower genetic map and saf flower draft genome assembly and genetic map showing the relative position of 2777 gene sequences. The genes from 50 syntenic segments highlighted in red contain 74% of all gene sequences on both maps.

Sequences from 6994 single copy, mapped sunflower genes (Bowers *et al.* 2012a) were compared to the safflower genome assembly, and 6419 (92%) could be placed on scaffolds within that assembly. Of these, 2777 (43%) matched genetically mapped scaffolds. Inspection of Figure 5 reveals a more complex pattern of synteny *vs.* the sunflower genome, with each region of the safflower genome corresponding to two different regions in sunflower. This pattern is due to a previously described polyploidy event that occurred near the base of the Heliantheae on the lineage leading to sunflower (Barker *et al.* 2008). In part due to this genome duplication, a large number of syntenic segments (50 in total) could be detected between the two species. These syntenic segments contained 74% of the 2777 sequences localized in both genomes. Once again, this result suggests that a higher than random fraction of the single copy gene sequences is present in the successfully mapped safflower scaffolds.

### Conclusions

The general approach outlined above for the sequence-based construction of genetic maps has the potential to efficiently produce maps containing 100–1000 × as many loci as most genetic maps produced in recent years. At the same time, this approach can also offer substantial cost and time savings over older genotyping technologies. However, due to difficulties associated with accurately identifying heterozygous genotypes when dealing with low coverage data, this method is best applied to highly homozygous mapping populations (*e.g.*, RILs or doubled haploids). Moreover, despite the large number of mapped loci, the resolution of such maps is still limited by the size of the mapping population. Despite these limitations, such maps can be powerful tools for comparative genomic analyses, assisting in the assembly of high quality genomes in otherwise uncharacterized species, and/or identifying candidate genes underlying previously mapped traits (Nambeesan *et al.* 2015). If more widely adopted, even minor crops could have better genetic mapping resources than were available in the most well-characterized species a few years ago.

## Supplementary Material

Supplemental Material
