# Supplementary material for: Genetic Mapping of Millions of SNPs in Safflower (Carthamus tinctorius L.) via Whole-Genome Resequencing
Source: G3 (Bethesda). 2016 May 24;6(7):2203–11. doi: 10.1534/g3.115.026690 (PMC4938673; doi:10.1534/g3.115.026690)
Supplement: Supplemental Material [file supp_6_7_2203__index.html]

Genetic Mapping of Millions of SNPs in Safflower (Carthamus tinctorius L.) via Whole Genome Resequencing — Genetic Mapping of Millions of SNPs in Safflower (Carthamus tinctorius L.) via Whole-Genome Resequencing — Supplemental Material 

# Genetic Mapping of Millions of SNPs in Safflower (*Carthamus tinctorius* L.) via Whole-Genome Resequencing

## Supplemental Material for Bowers, Pearl, and Burke, 2016

**Files in this Data Supplement:**

- Figure S1 - Each panel depicts the number of base pairs, SNPs, and scaffolds assigned to each centimorgan (cM) position for a given safflower linkage group. (.pdf, 384 KB)
- File S1 - Example of genetic mapping using spreadsheet software. (.pptx, 884 KB)
- File S2 - Fasta file of draft safflower genome. (.zip, 264 MB)
- File S3 - Statistics on draft genome assembly. (.txt, 2 KB)
- File S4 - Safflower genetic map - Recombination bins. (.xlsx, 6 MB)
- File S5 - Safflower genetic map - Consensus genotypes and map positions of sequence contigs. (.xlsx, 27 MB)
- File S6 - Raw data genotypes of individual SNPs. (.csv, 704 MB)
