# Supplementary material for: Genetic Mapping of Millions of SNPs in Safflower (Carthamus tinctorius L.) via Whole-Genome Resequencing
Source: G3 (Bethesda). 2016 May 24;6(7):2203–11. doi: 10.1534/g3.115.026690 (PMC4938673; doi:10.1534/g3.115.026690)
Supplement: Supplemental Material [file supp_g3.115.026690_FigureS1.pdf]

Figure S1. Each panel depicts the number of base pairs, SNPs, and scaffolds assigned to each centimorgan (cM) position for a given safflower linkage group.

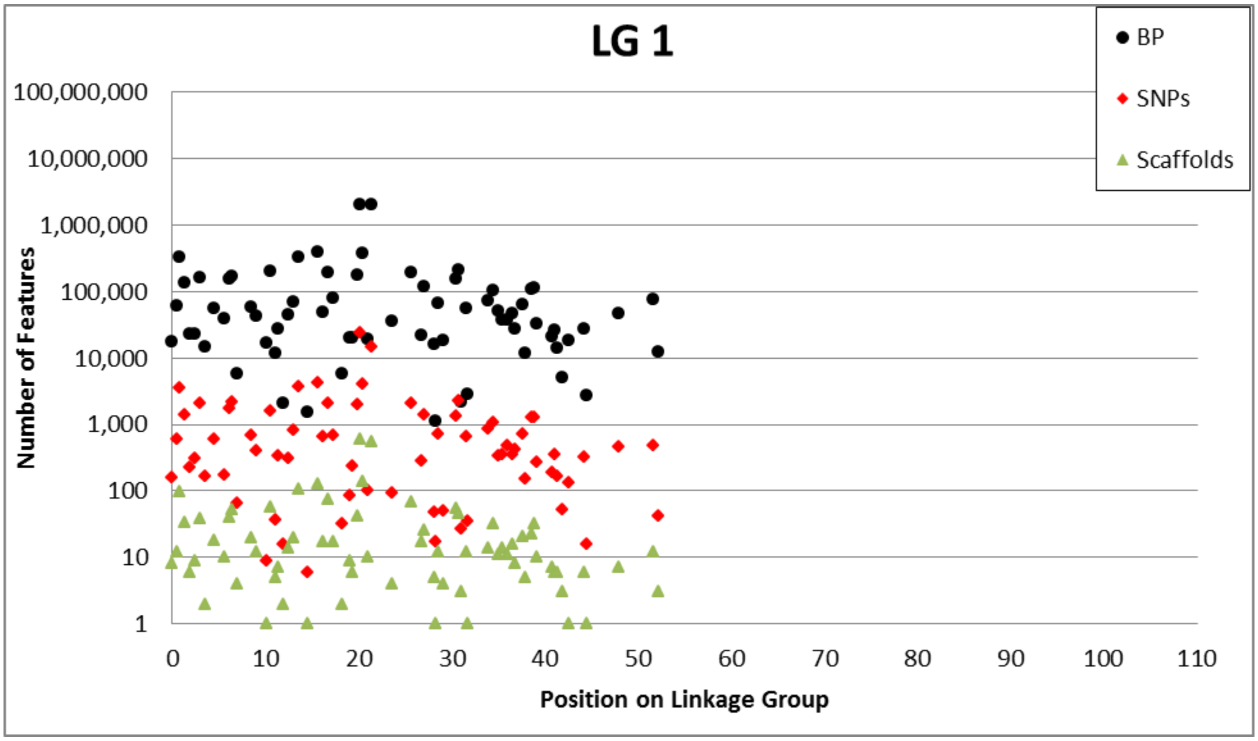

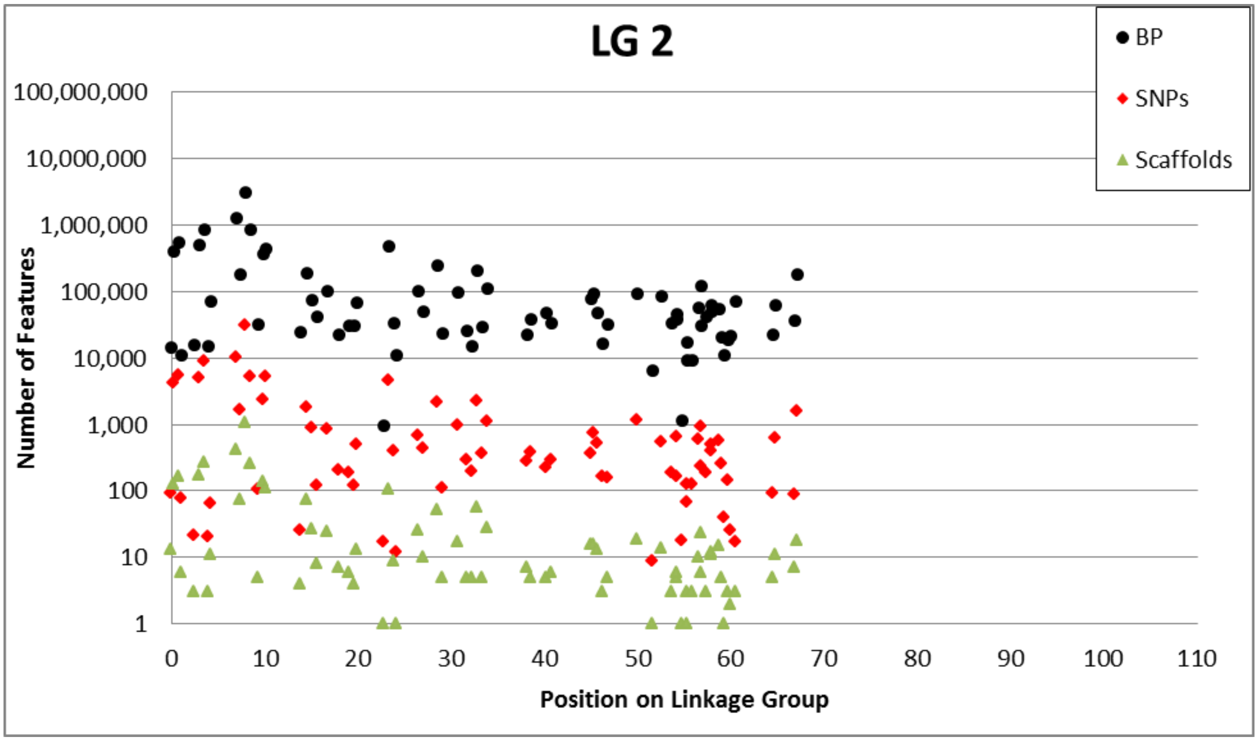

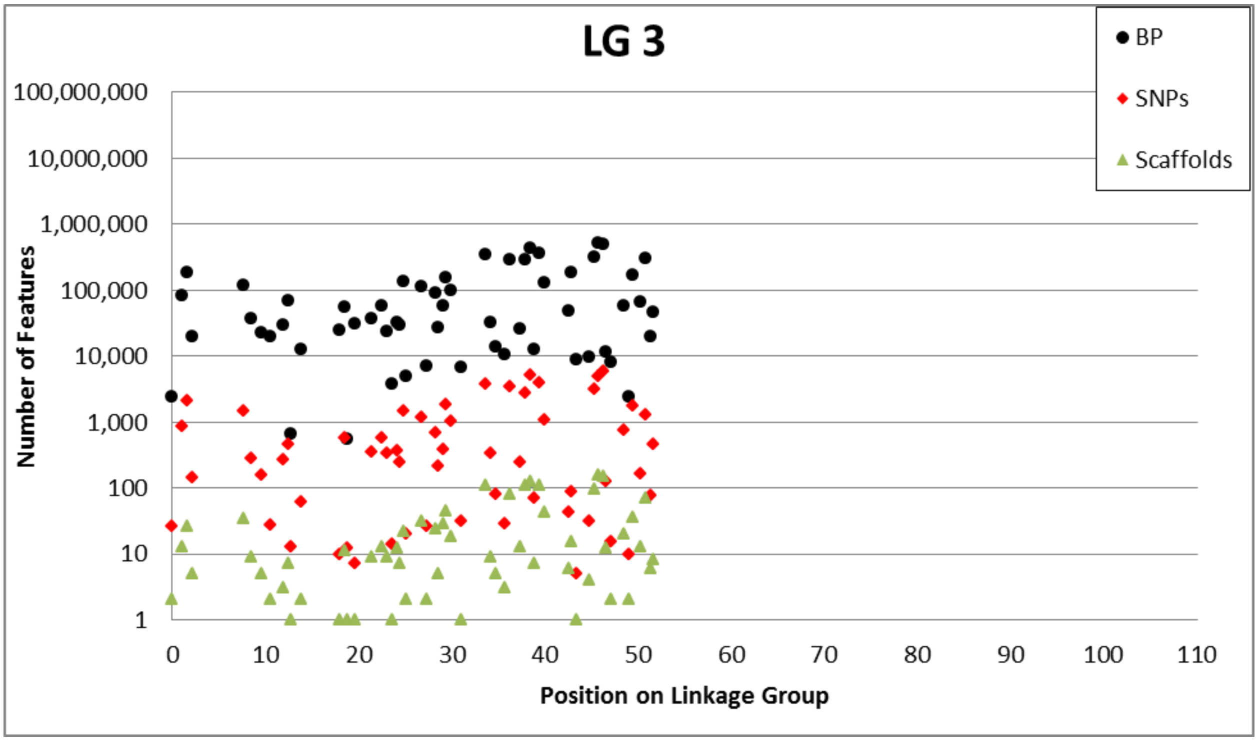
LG 3
BP
SNPs
Scaffolds
100,000,000
10,000,000
1,000,000
100,000
10,000
1,000
100
10
1
Number of Features
0
10
20
30
40
50
60
70
80
90
100
110
Position on Linkage Group

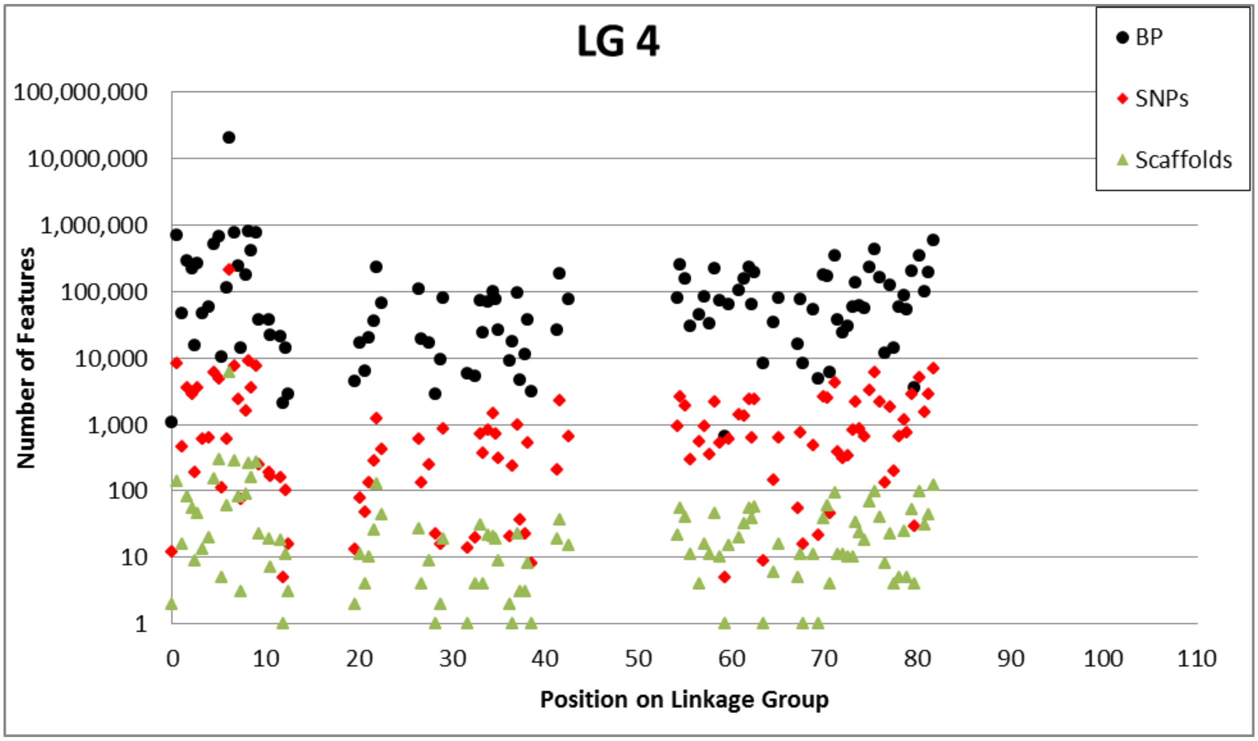
LG 4
BP
SNPs
Scaffolds
100,000,000
10,000,000
1,000,000
100,000
10,000
1,000
100
10
1
Number of Features
0
10
20
30
40
50
60
70
80
90
100
110
Position on Linkage Group

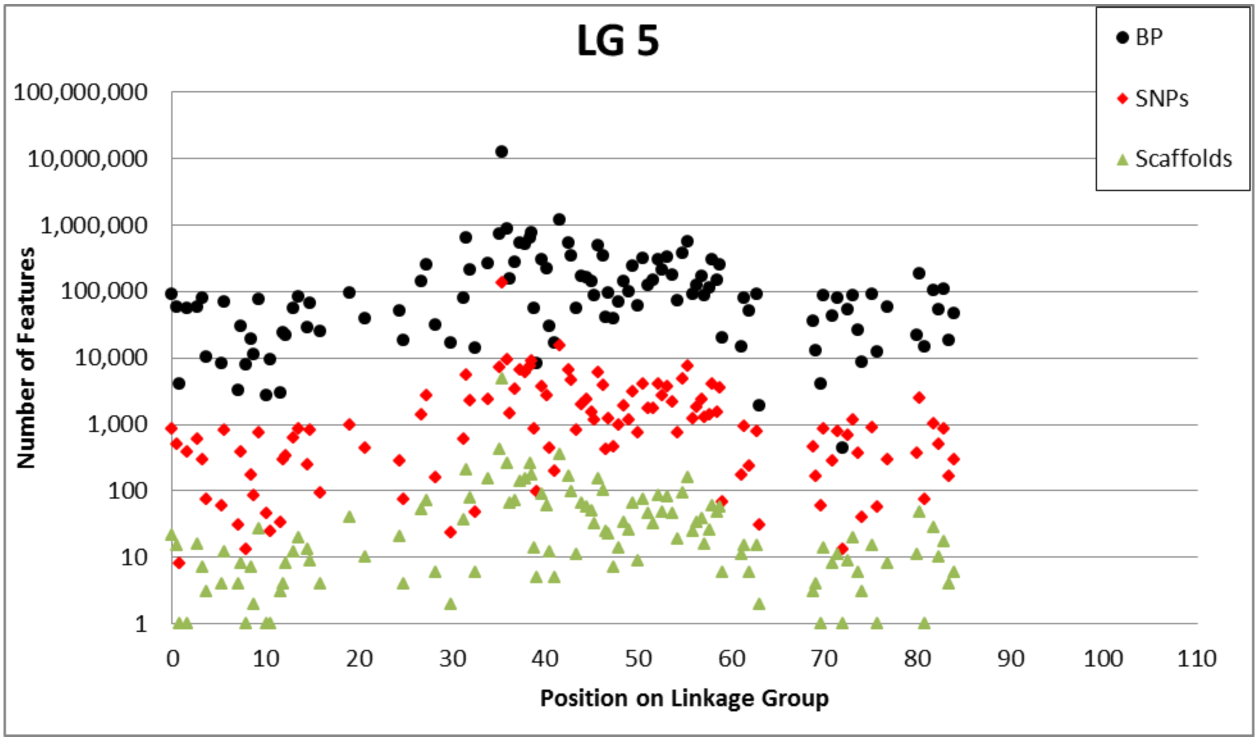
LG 5
BP
SNPs
Scaffolds
100,000,000
10,000,000
1,000,000
100,000
10,000
1,000
100
10
1
Number of Features
0
10
20
30
40
50
60
70
80
90
100
110
Position on Linkage Group

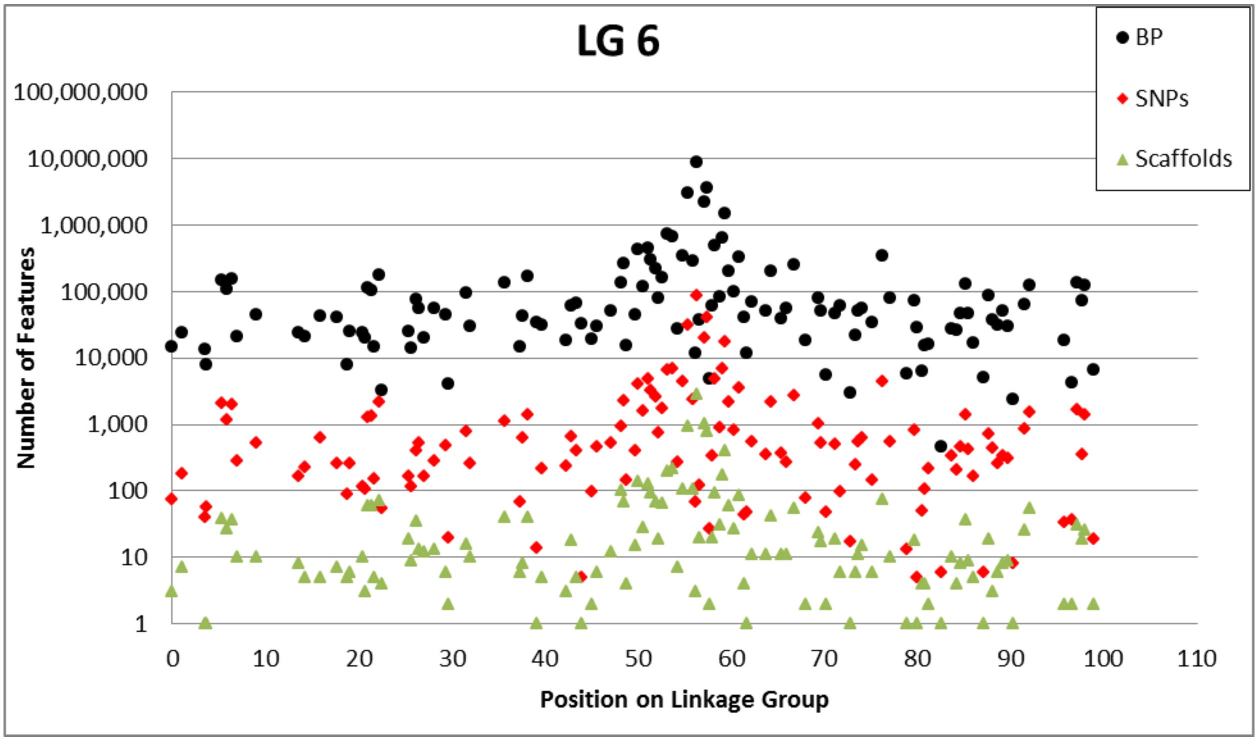
LG 6
BP
SNPs
Scaffolds
100,000,000
10,000,000
1,000,000
100,000
10,000
1,000
100
10
1
Number of Features
0
10
20
30
40
50
60
70
80
90
100
110
Position on Linkage Group

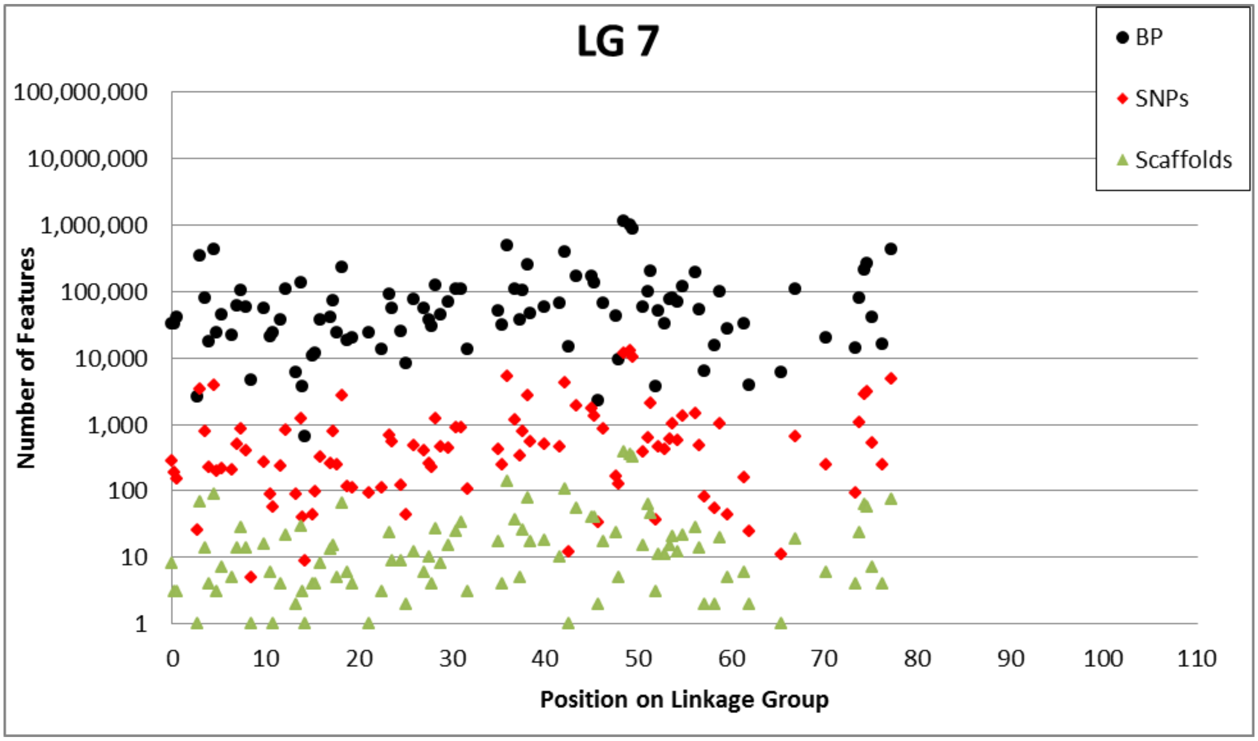
LG 7
BP
SNPs
Scaffolds
100,000,000
10,000,000
1,000,000
100,000
10,000
1,000
100
10
1
Number of Features
0
10
20
30
40
50
60
70
80
90
100
110
Position on Linkage Group

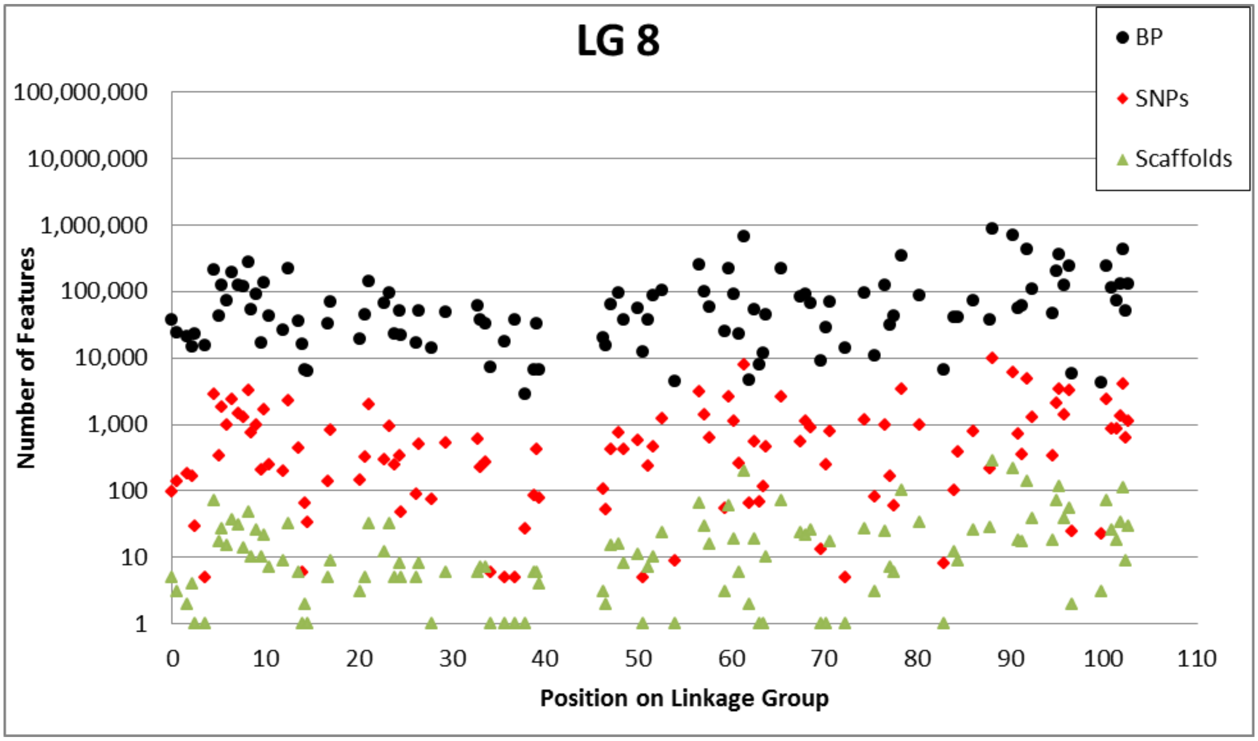
LG 8
BP
SNPs
Scaffolds
100,000,000
10,000,000
1,000,000
100,000
10,000
1,000
100
10
1
Number of Features
0
10
20
30
40
50
60
70
80
90
100
110
Position on Linkage Group

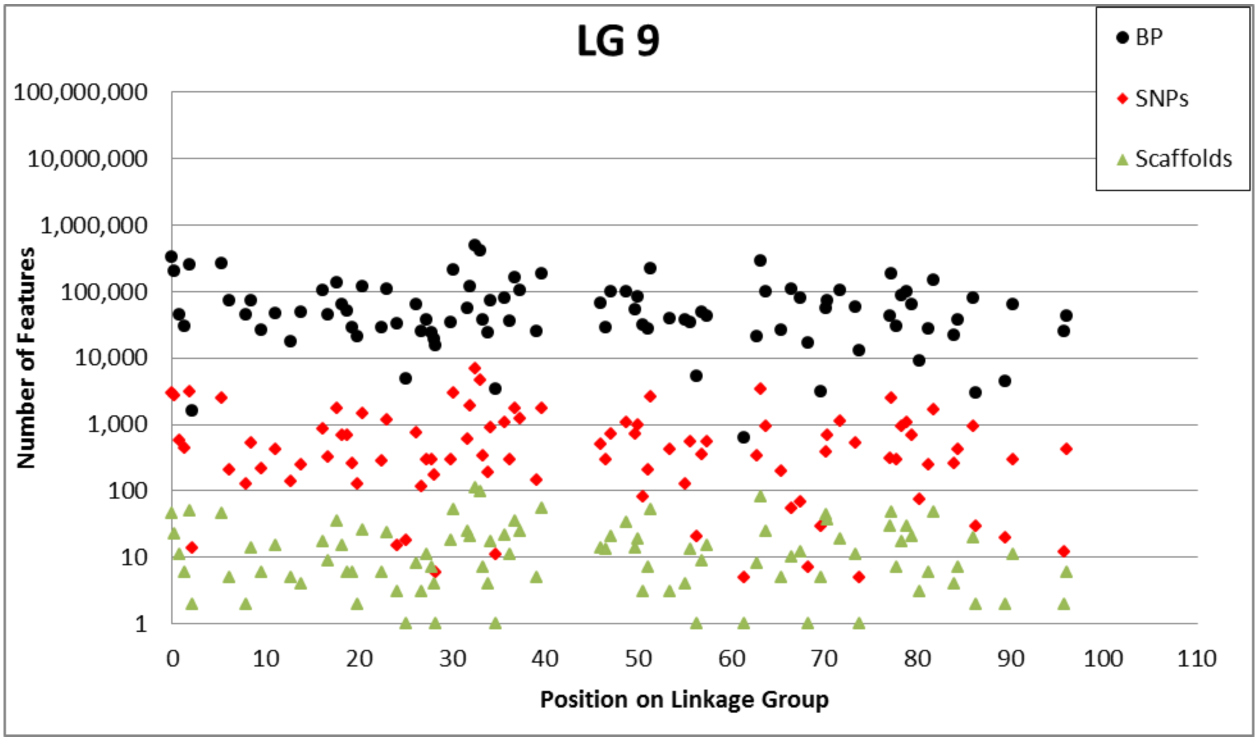
LG 9
BP
SNPs
Scaffolds
100,000,000
10,000,000
1,000,000
100,000
10,000
1,000
100
10
1
Number of Features
0
10
20
30
40
50
60
70
80
90
100
110
Position on Linkage Group

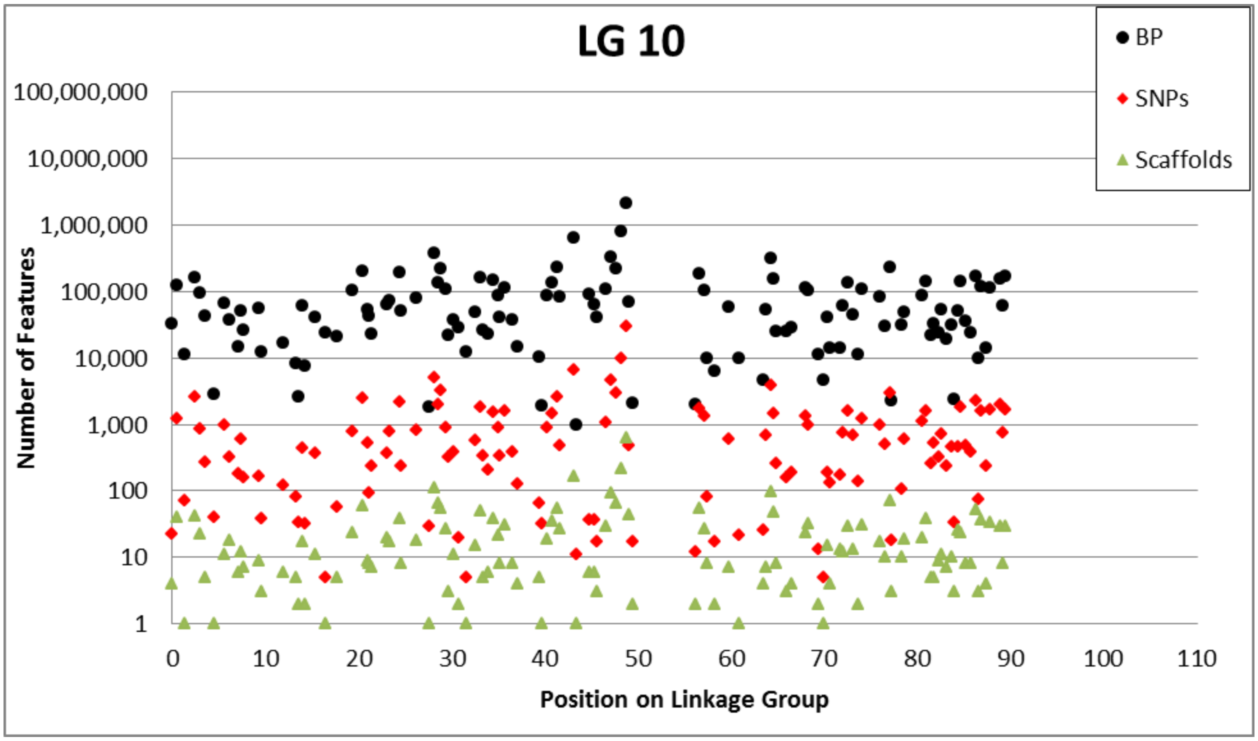
LG 10
BP
SNPs
Scaffolds
100,000,000
10,000,000
1,000,000
100,000
10,000
1,000
100
10
1
Number of Features
0
10
20
30
40
50
60
70
80
90
100
110
Position on Linkage Group

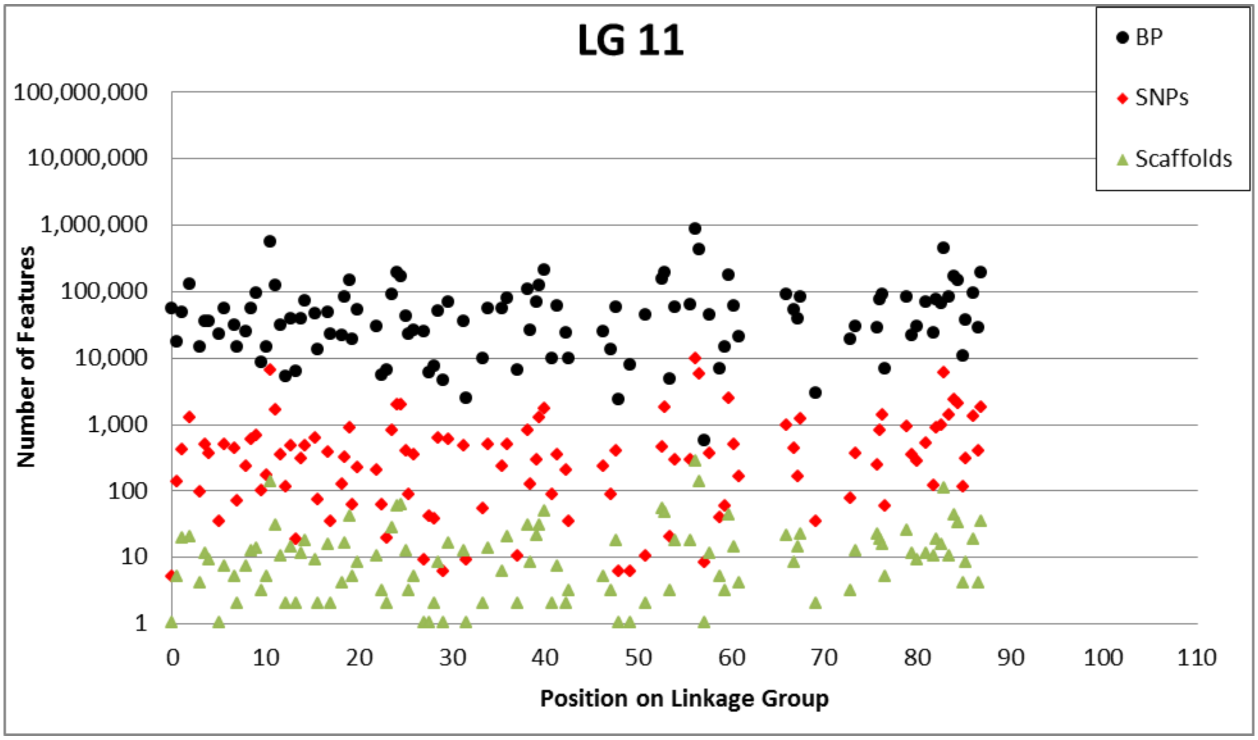
LG 11
BP
SNPs
Scaffolds
100,000,000
10,000,000
1,000,000
100,000
10,000
1,000
100
10
1
Number of Features
0
10
20
30
40
50
60
70
80
90
100
110
Position on Linkage Group

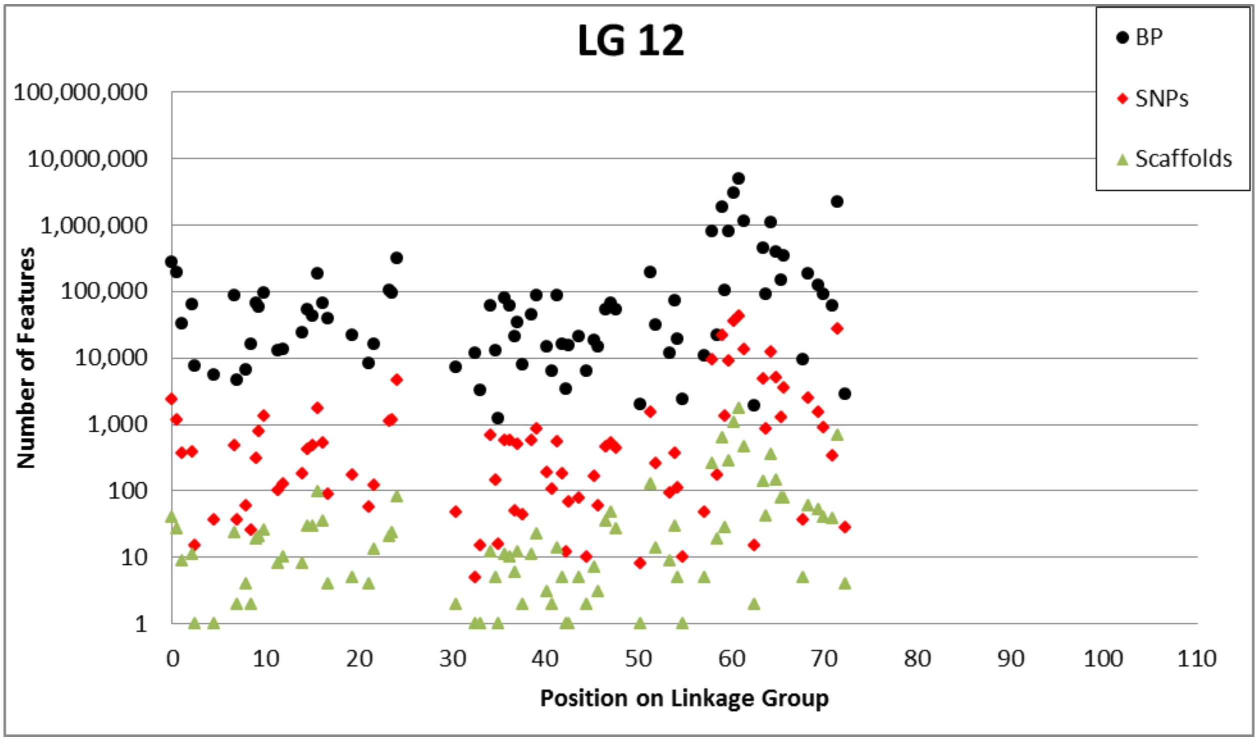
LG 12
BP
SNPs
Scaffolds
100,000,000
10,000,000
1,000,000
100,000
10,000
1,000
100
10
1
Number of Features
0
10
20
30
40
50
60
70
80
90
100
110
Position on Linkage Group
