# Supplementary material for: Genetic Mapping of Millions of SNPs in Safflower (Carthamus tinctorius L.) via Whole-Genome Resequencing
Source: G3 (Bethesda). 2016 May 24;6(7):2203–11. doi: 10.1534/g3.115.026690 (PMC4938673; doi:10.1534/g3.115.026690)
Supplement: Supplemental Material [file supp_g3.115.026690_FileS1.pptx]

## Slide 1
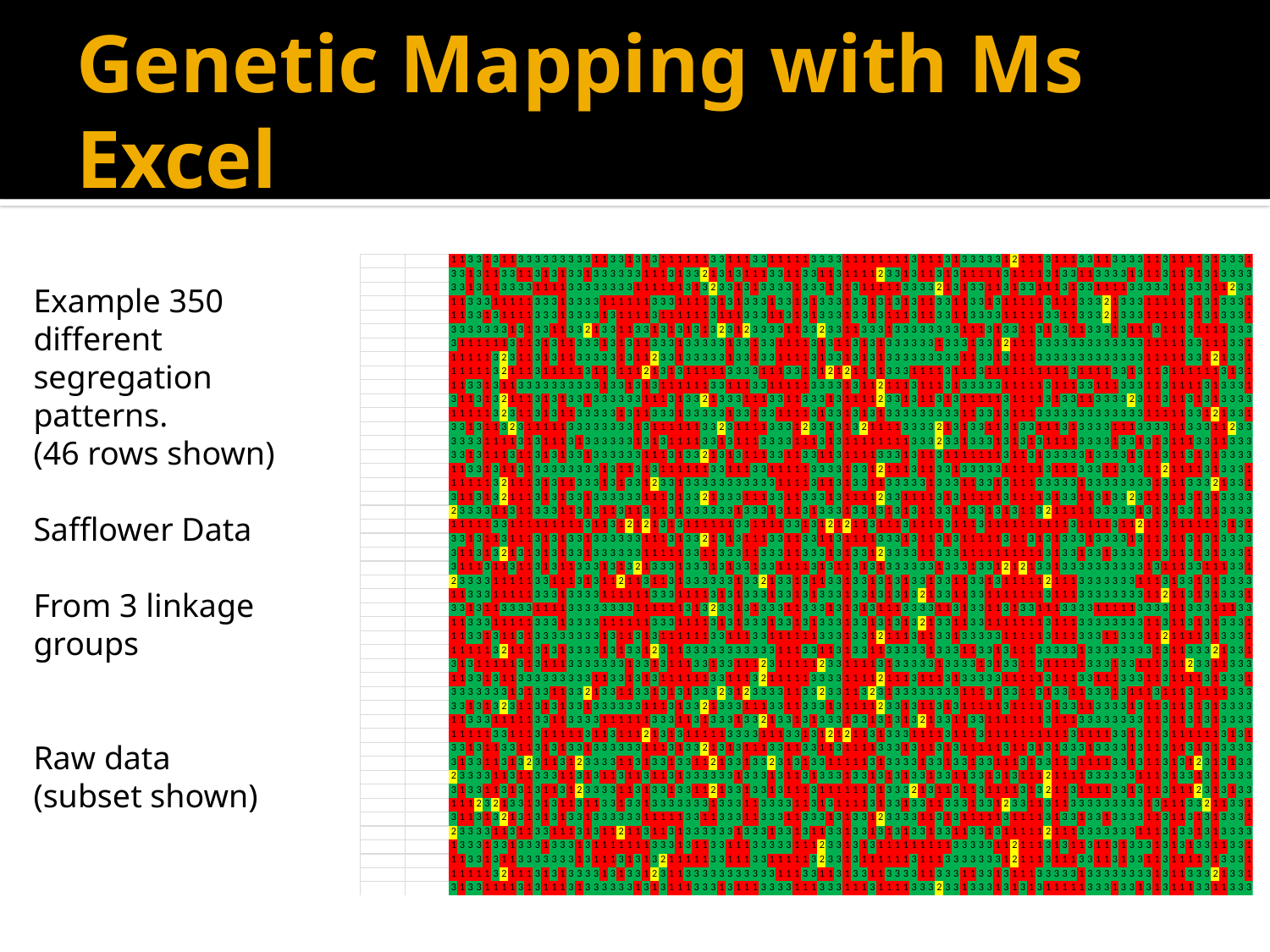

# Genetic Mapping with Ms Excel
Example 350 different segregation patterns.
(46 rows shown)
Safflower Data
From 3 linkage groups
Raw data
(subset shown)

## Slide 2
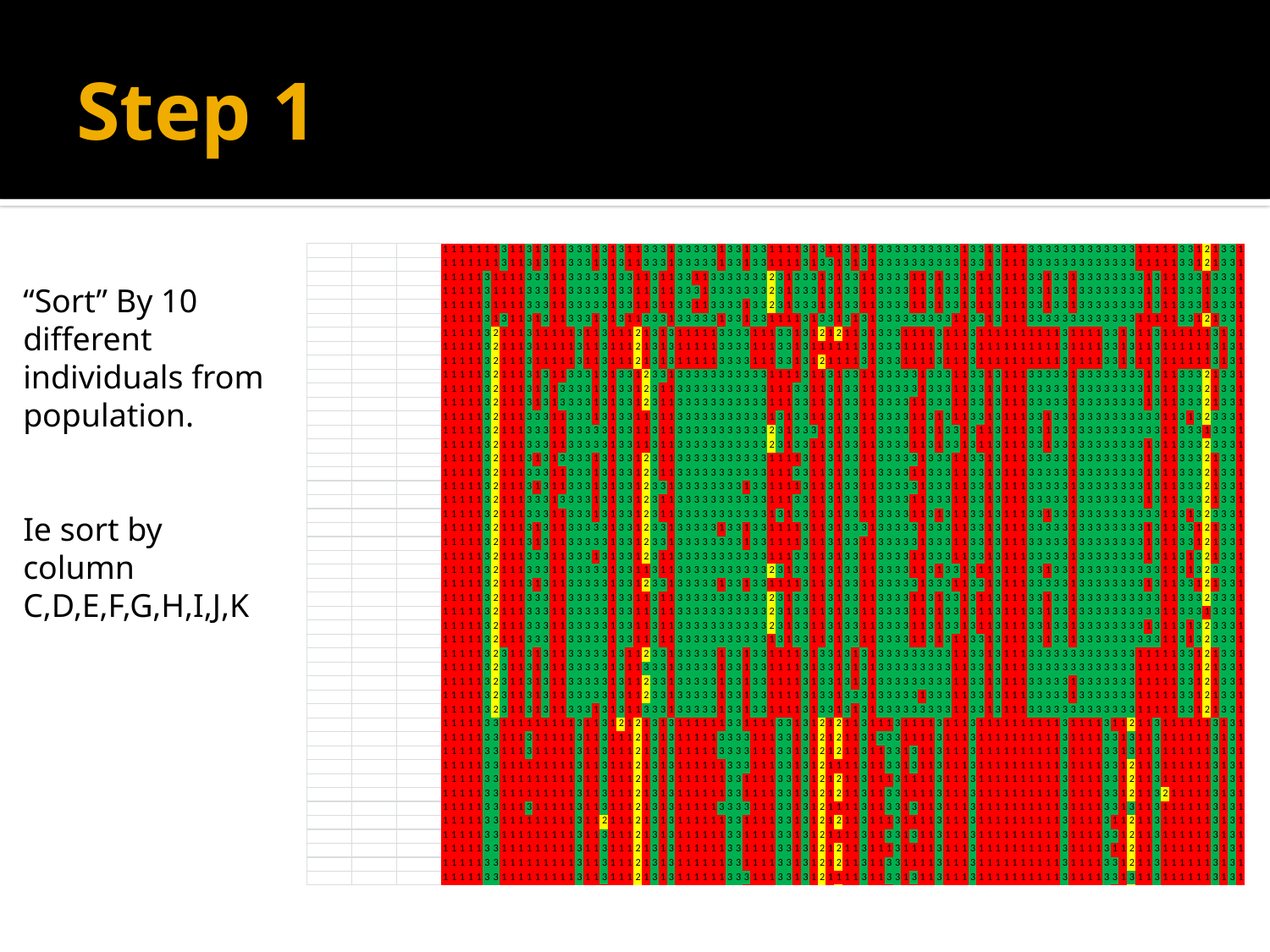

# Step 1
“Sort” By 10 different individuals from population.
Ie sort by column C,D,E,F,G,H,I,J,K

## Slide 3
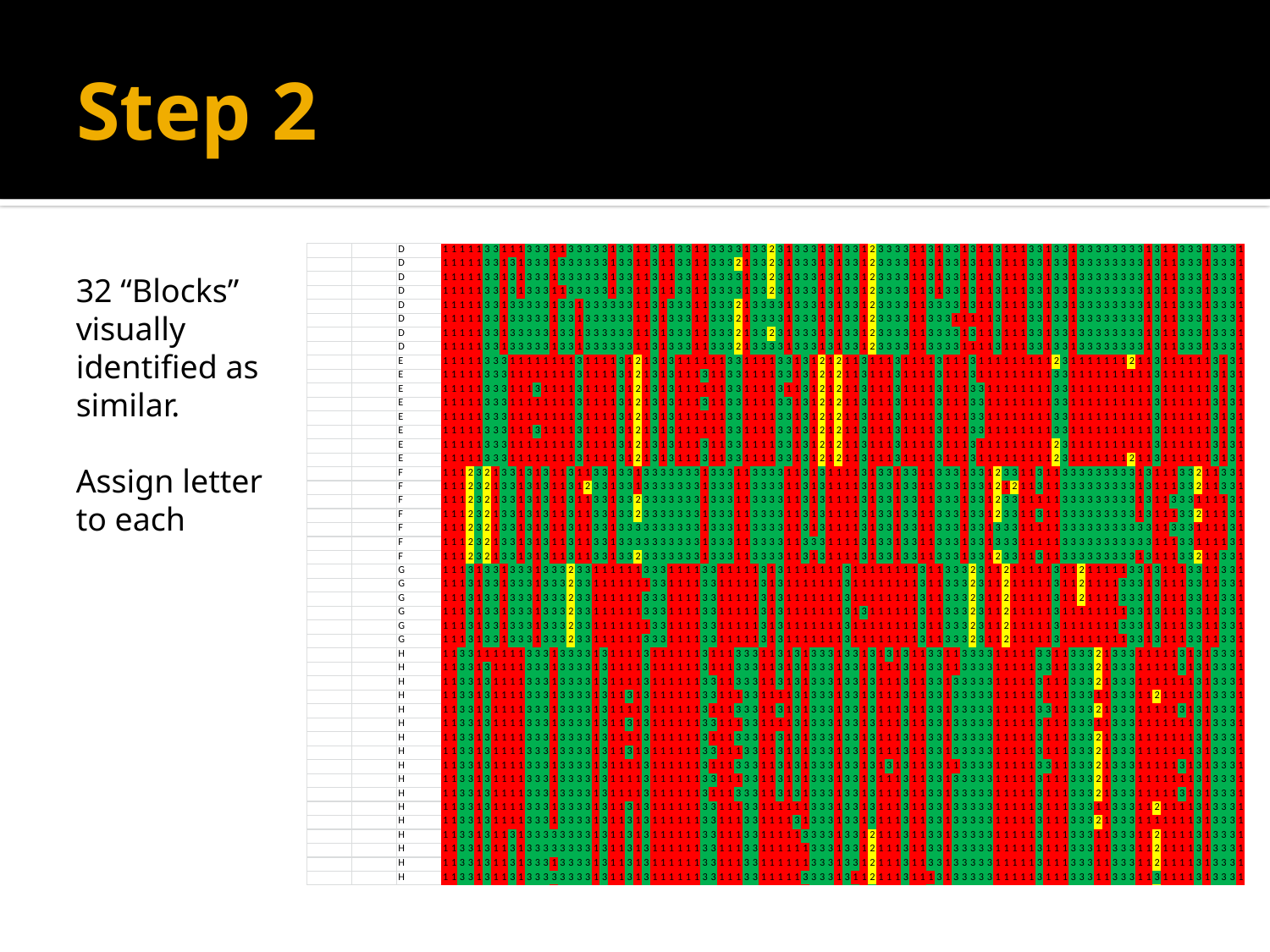

# Step 2
32 “Blocks” visually identified as similar.
Assign letter to each

## Slide 4
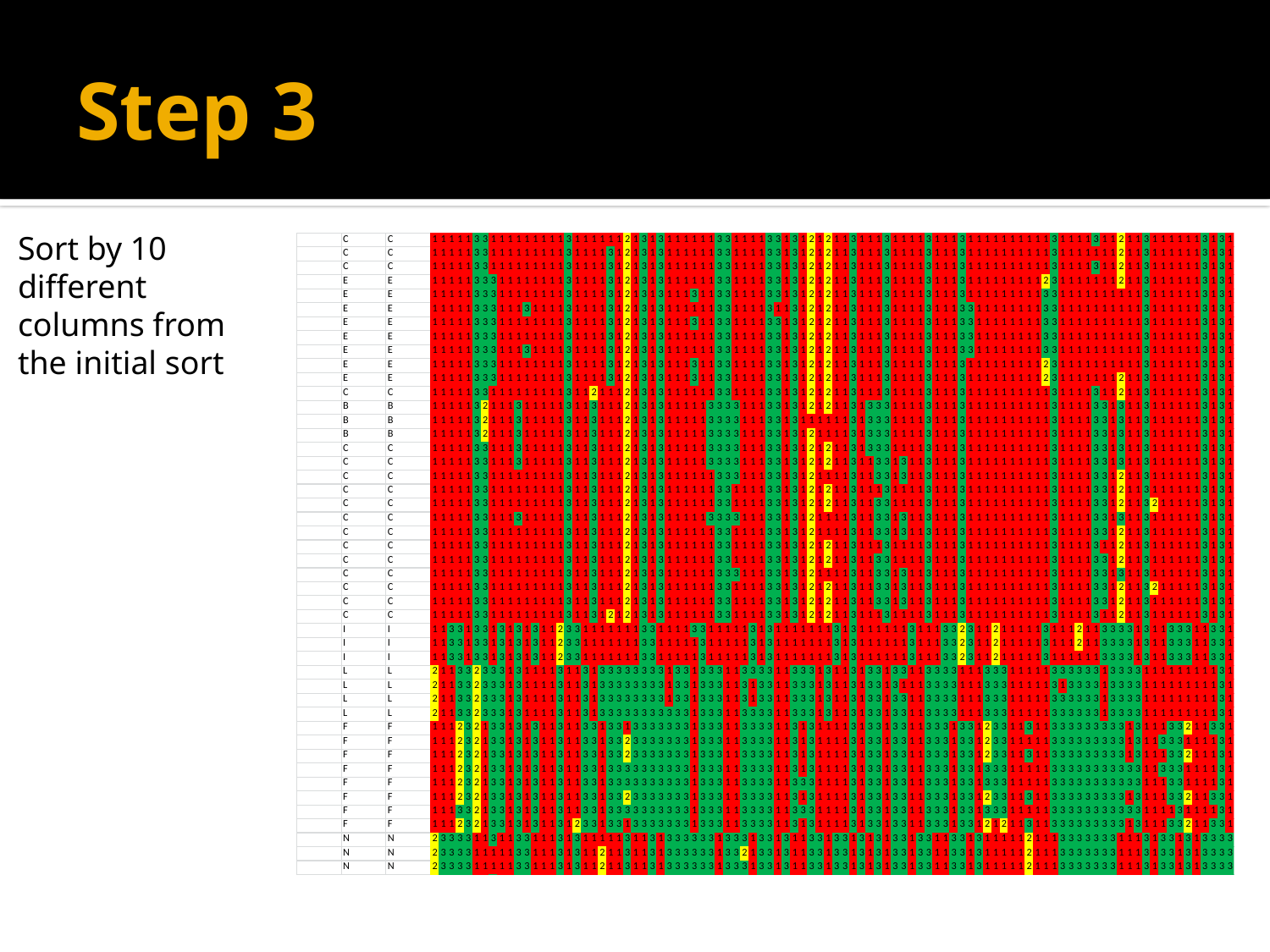

# Step 3
Sort by 10 different columns from the initial sort

## Slide 5
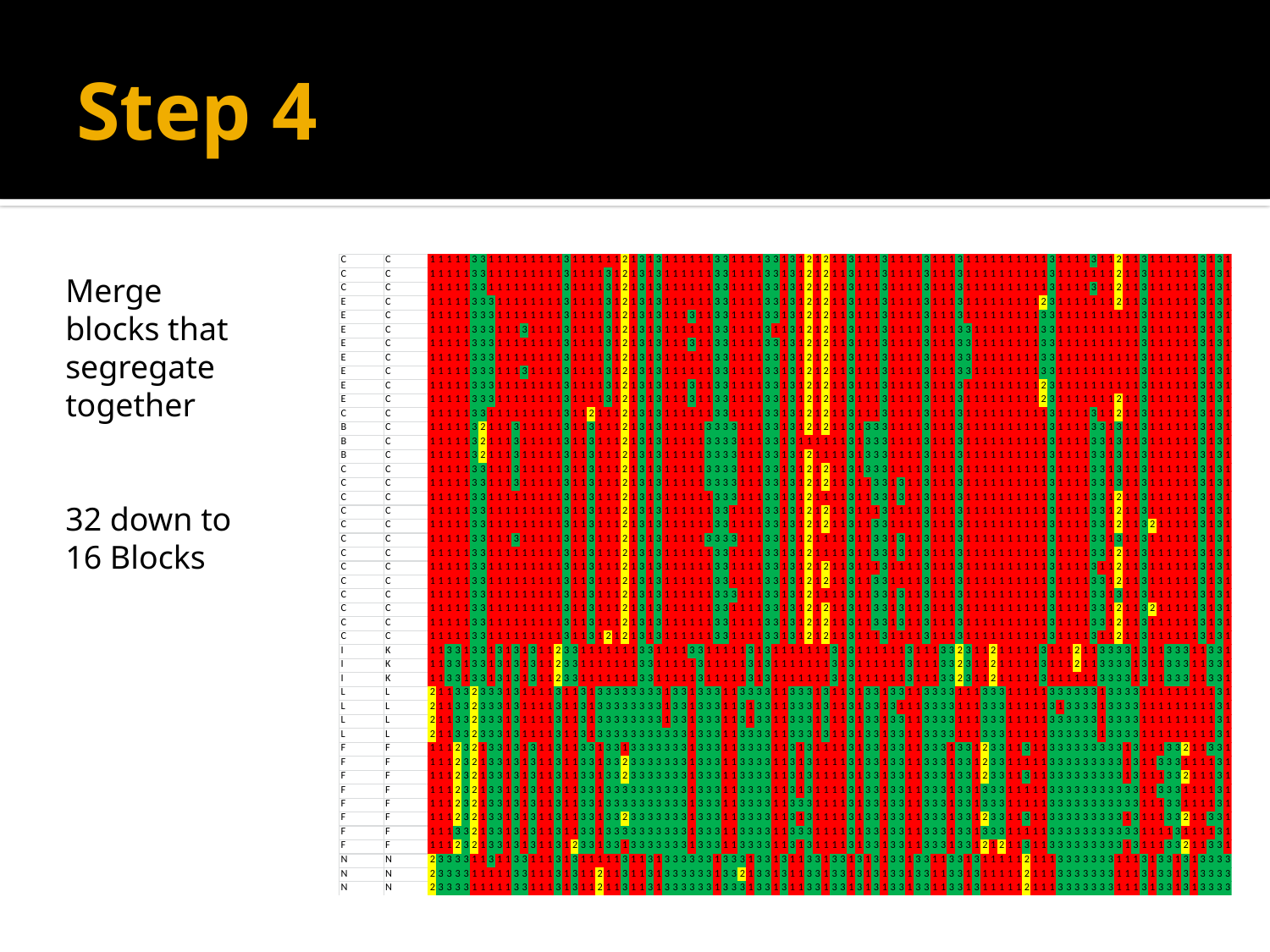

# Step 4
Merge blocks that segregate together
32 down to 16 Blocks

## Slide 6
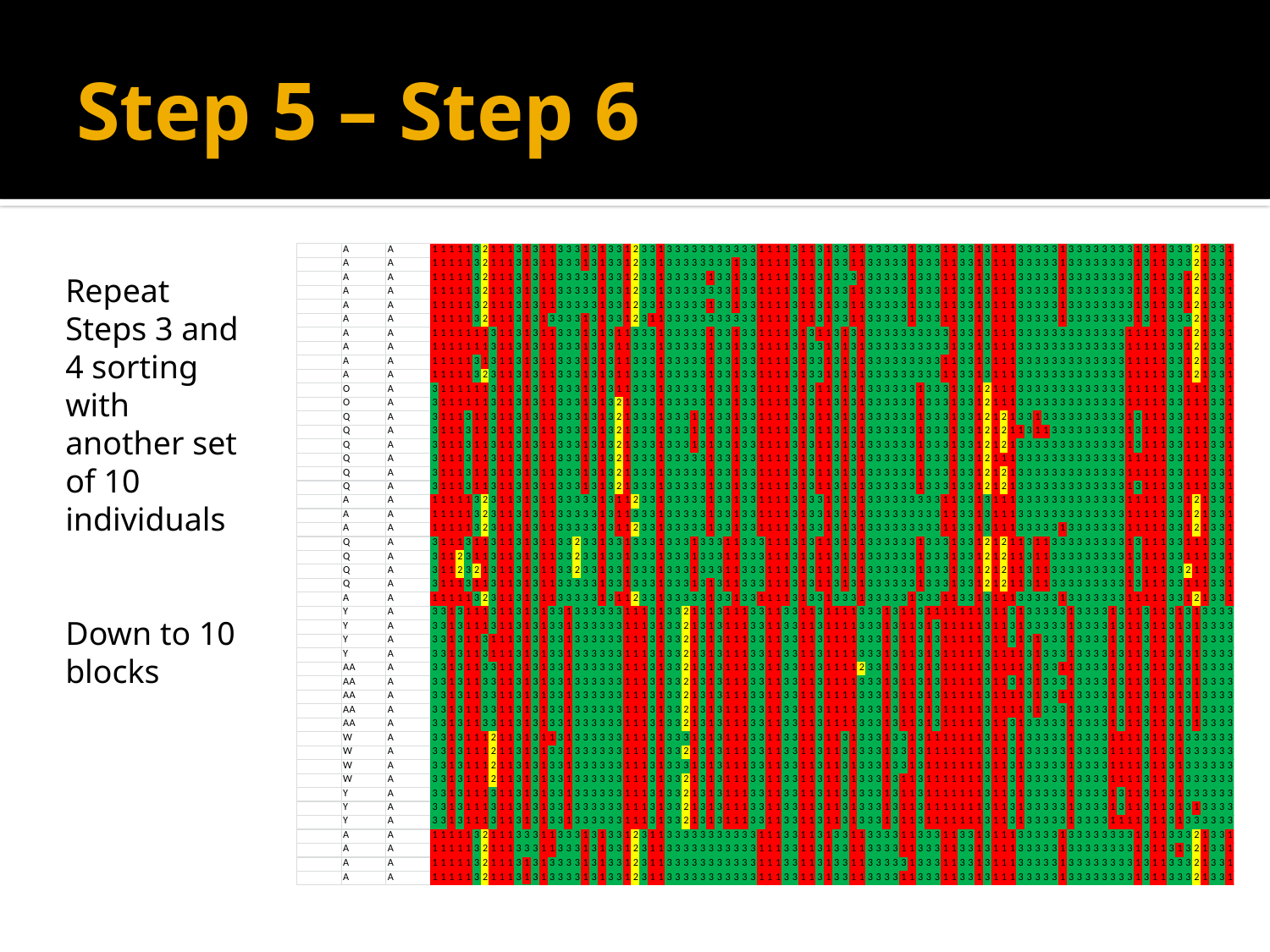

# Step 5 – Step 6
Repeat Steps 3 and 4 sorting with another set of 10 individuals
Down to 10 blocks

## Slide 7
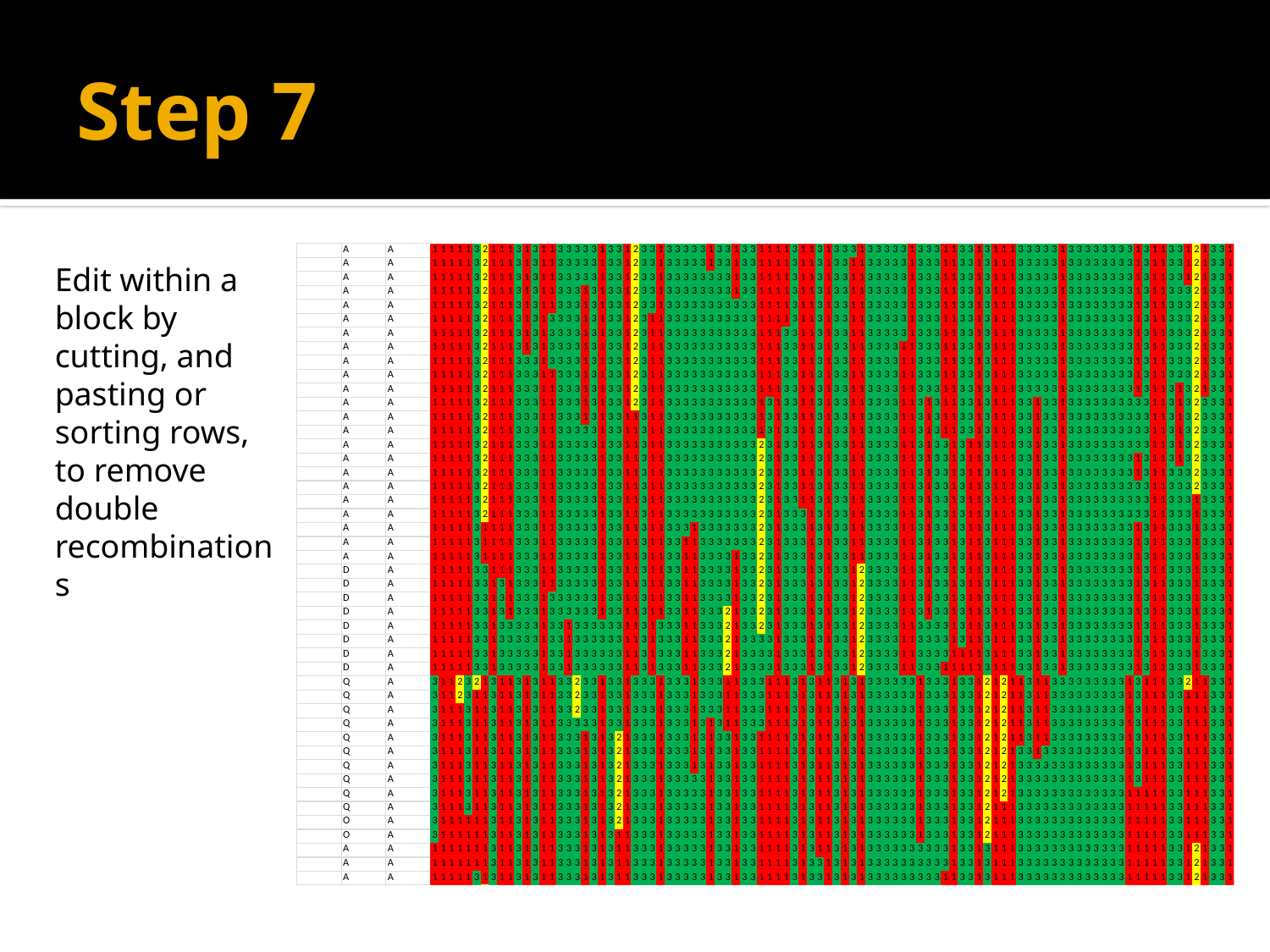

# Step 7
Edit within a block by cutting, and pasting or sorting rows, to remove double recombinations

## Slide 8
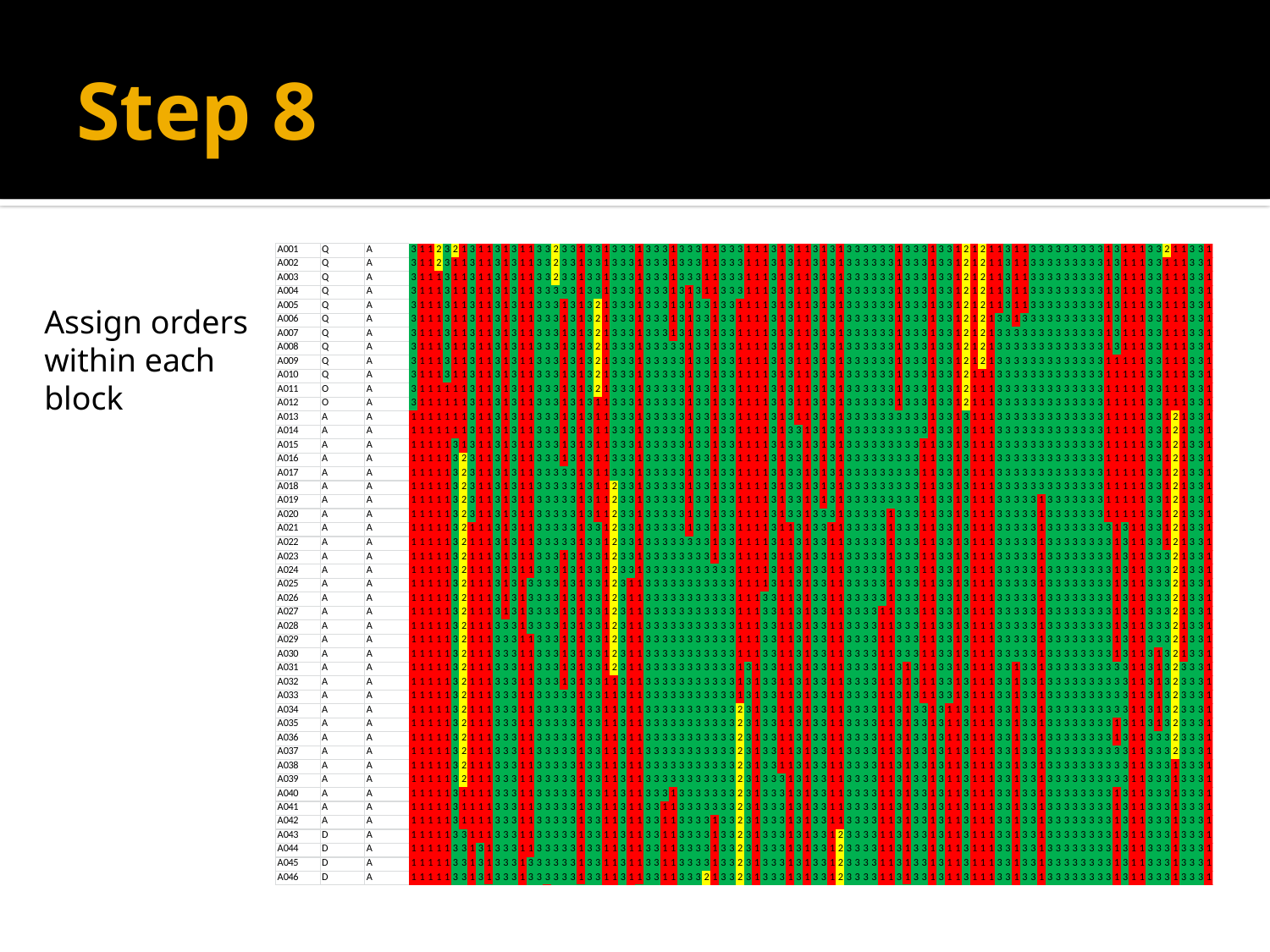

# Step 8
Assign orders within each block

## Slide 9
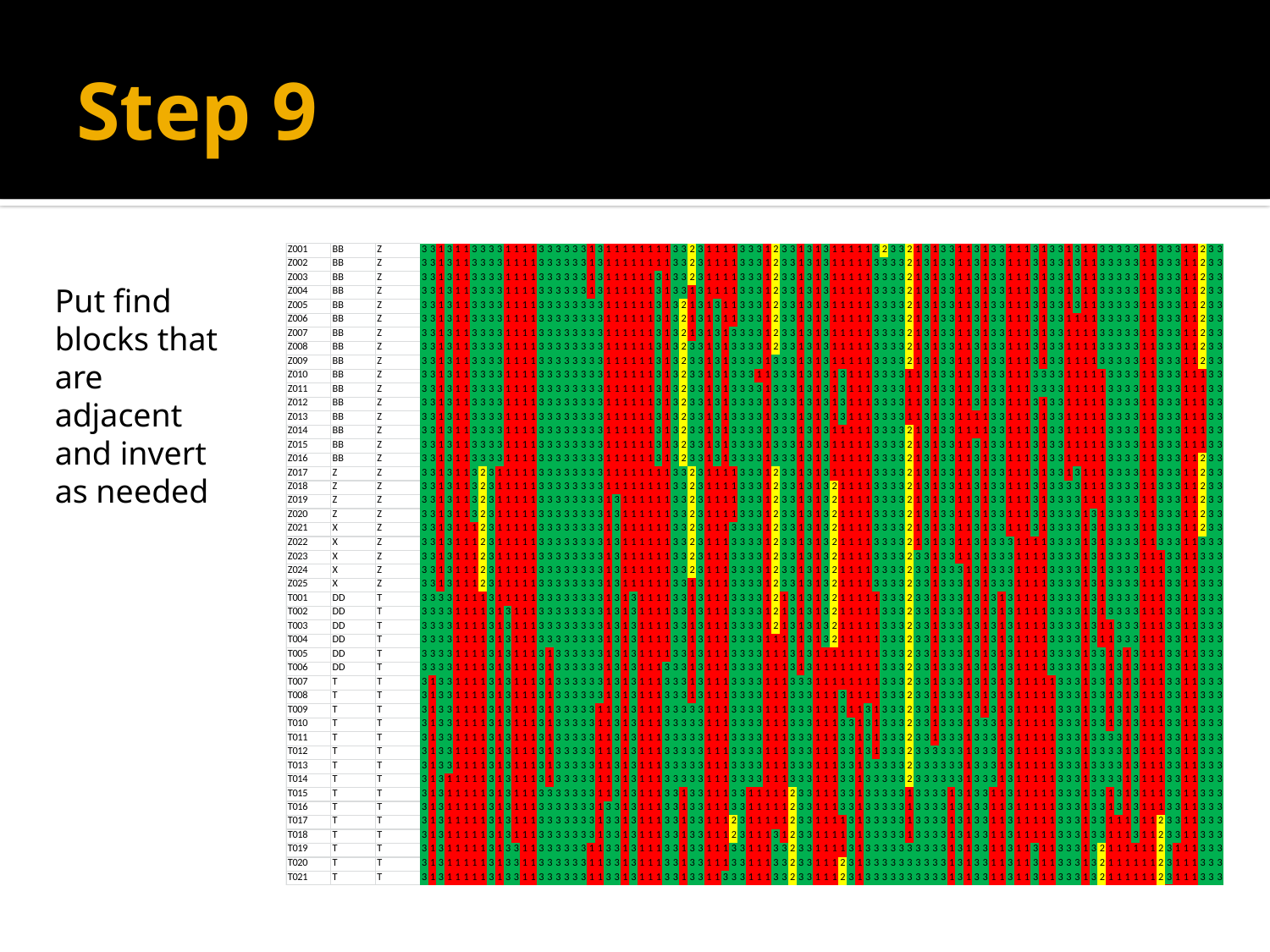

# Step 9
Put find blocks that are adjacent and invert as needed

## Slide 10
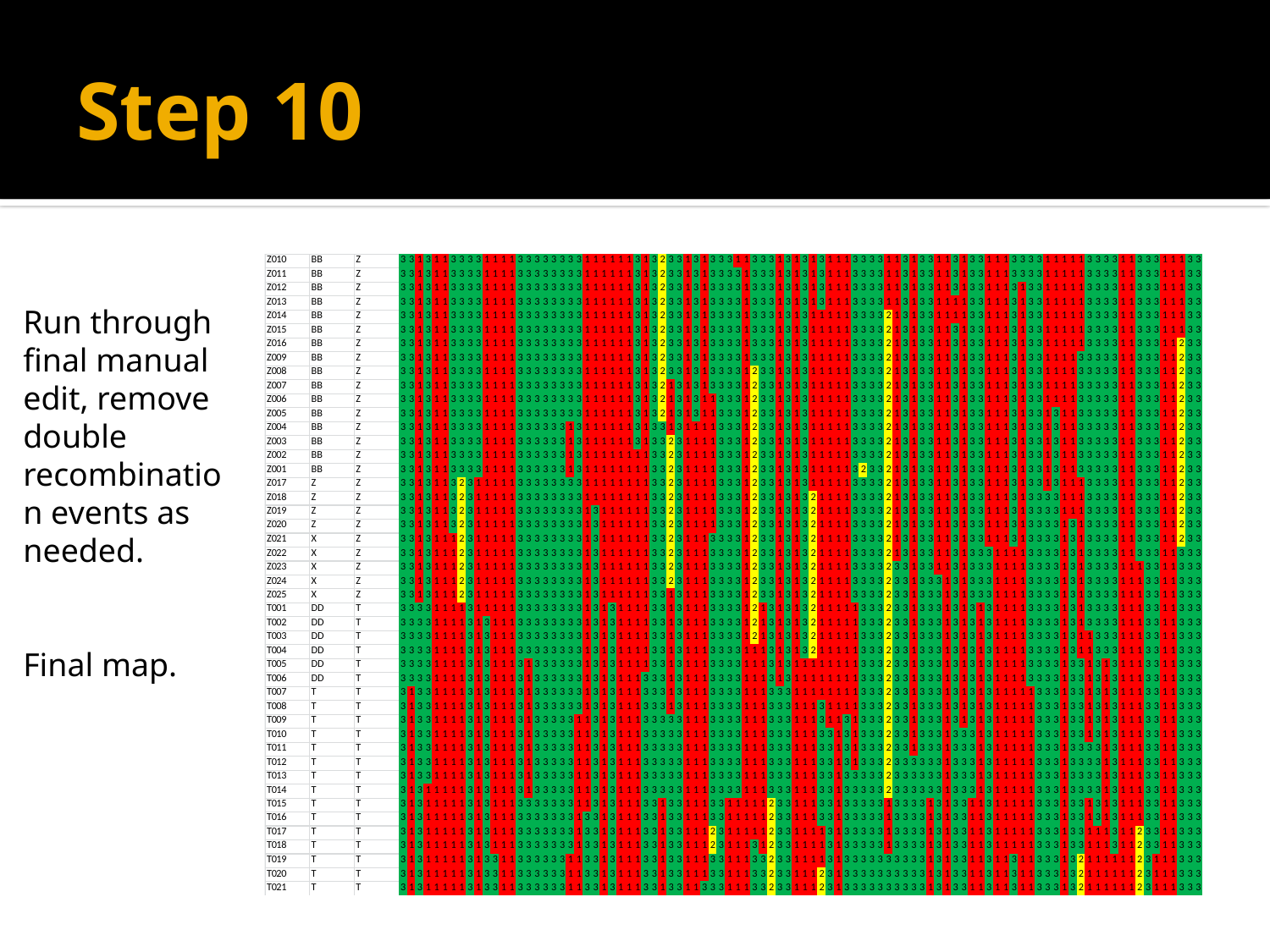

# Step 10
Run through final manual edit, remove double recombination events as needed.
Final map.
